# RNA demethylase ALKBH5 promotes tumorigenesis in multiple myeloma via TRAF1-mediated activation of NF-κB and MAPK signaling pathways

**DOI:** 10.1038/s41388-021-02095-8

**Published:** 2021-11-10

**Authors:** Jianwei Qu, Yifan Hou, Qingxiao Chen, Jing Chen, Yi Li, Enfan Zhang, Huiyao Gu, Ruyi Xu, Yang Liu, Wen Cao, Jinna Zhang, Liqin Cao, Jingsong He, Zhen Cai

**Affiliations:** 1 Bone Marrow Transplantation Center, The First Affiliated Hospital, School of Medicine, Zhejiang University, Hangzhou, Zhejiang China; 2 Institute of Hematology, Zhejiang University, Hangzhou, Zhejiang China

**Keywords:** Myeloma, Oncogenes, Prognostic markers

## Abstract

N^6^-methyladenosine (m^6^A), an internal modification in mRNA, plays a critical role in regulating gene expression. Dysregulation of m^6^A modifiers promotes oncogenesis through enzymatic functions that disrupt the balance between the deposition and removal of m^6^A modification on critical transcripts. However, the roles of mRNA m^6^A in multiple myeloma (MM) are poorly understood. The present study showed that RNA demethylase ALKBH5 was overexpressed in MM and associated with a poor prognosis in MM patients. Knocking down *ALKBH5* induced apoptosis and inhibited the growth of MM cells in vitro. Xenograft models and gene set enrichment analysis with patient transcriptome datasets also supported the oncogenic role of ALKBH5 in MM. Mechanistic studies showed that ALKBH5 exerted tumorigenic effects in myeloma in an m^6^A-dependent manner, and TNF receptor-associated factor 1 (TRAF1) was a critical target of ALKBH5. Specifically, ALKBH5 regulated TRAF1 expression via decreasing m^6^A abundance in the 3'-untranslated region (3'-UTR) of TRAF1 transcripts and enhancing *TRAF1* mRNA stability. As a result, ALKBH5 promoted MM cell growth and survival through TRAF1-mediated activation of NF-κB and MAPK signaling pathways. Collectively, our data demonstrated that ALKBH5 played a critical role in MM tumorigenesis and suggested that ALKBH5 could be a novel therapeutic target in MM.

## Introduction

Multiple myeloma (MM) is a hematological malignancy characterized by uncontrolled expansion of monoclonal plasma cells in the bone marrow (BM) [1]. The malignant development of MM is associated with the accumulation of acquired genetic events and additional epigenetic alterations [2–5]. The disease remains incurable despite the progress made in MM treatments (for example, proteasome inhibitors, immunomodulatory drugs, and autologous stem cell transplantation) [6, 7]. New therapies with novel mechanisms of action are needed for patients with MM, especially those with refractory and high-risk myeloma [1, 6, 7]. Thus, exploring distinct molecular mechanisms underlying myeloma tumorigenesis is essential to develop potentially effective therapeutic agents.

N^6^-methyladenosine (m^6^A) is a prevalent and abundant chemical modification in messenger RNA (mRNA) in eukaryotes [8, 9]. The methylation of N^6^-adenosine is mainly catalyzed by m^6^A methyltransferase (writer) complex composed of methyltransferase-like 3 (METTL3), methyltransferase-like 14 (METTL14), and Wilms’s tumor 1-associating protein (WTAP) [10, 11]. As a reversible modification, m^6^A methylation can be removed by m^6^A demethylases (erasers), including fat mass and obesity-associated protein (FTO) and α-ketoglutarate-dependent dioxygenase alkB homolog 5 (ALKBH5) [12, 13]. The dynamic m^6^A modification of mRNAs can be recognized by various m^6^A-binding proteins (readers), leading to diverse alterations in mRNA metabolisms, such as mRNA stability [14–16], translation efficiency [17, 18], and alternative splicing [19]. Accumulating evidence indicated that the m^6^A modification and its modulators (writers, erasers, and readers) are involved in the pathological processes in various cancers [20, 21]. In MM, the m^6^A reader protein heterogeneous nuclear ribonucleoproteins A2/B1 (HNRNPA2B1) plays a pivotal role in promoting MM progression by upregulating AKT3 expression through m^6^A-dependent stabilization of *ILF3* mRNA [22]. However, the functions of mRNA m^6^A methylation in MM have not yet been investigated in depth. Especially, the roles of m^6^A writers and erasers in MM development remain largely unknown.

ALKBH5 is a 2-oxoglutarate (2OG) and ferrous iron-dependent nucleic acid oxygenase [23, 24]. Soon after its identification as an m^6^A eraser [12], ALKBH5 was found to be involved in the initiation and progression of various cancers. Zhang et al. demonstrated that high ALKBH5 expression in glioblastoma (GBM) promotes the proliferation of GBM stem-like cells by sustaining FOXM1 expression [25]. In acute myeloid leukemia (AML), ALKBH5 was required to maintain leukemia stem cell self-renewal and promote AML tumorigenesis by regulating the expression of a set of critical genes (*AXL* and *TACC3*) at the posttranscriptional level [26, 27]. Conversely, ALKBH5 also served as a tumor suppressor in some malignancies. For example, Chen et al. found that ALKBH5 exhibited a tumor-suppressive role by inhibiting the expression of LYPD1 in hepatocellular carcinoma cells [28]. These studies suggested that the aberrant expression of key genes caused by the dysregulation of ALKBH5 led to a significant phenotypic change in specific cancers. However, the roles and underlying mechanisms of ALKBH5 in MM are not yet reported. Hence, we conducted a series of functional and mechanistic studies, which indicated a critical role of ALKBH5 in MM tumorigenesis as an m^6^A demethylase, thereby suggesting that ALKBH5 is a promising therapeutic target in MM.

## Results

### Increased ALKBH5 expression was associated with poor prognosis in MM

**Fig. 1:**
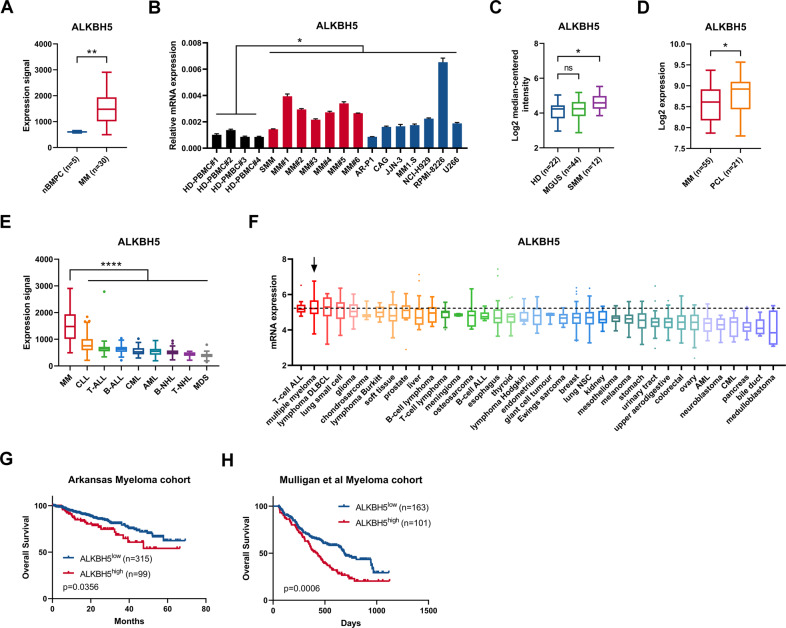
ALKBH5 overexpression was associated with poor survival of patients with MM. **A** Normalized expression signal of ALKBH5 in nBMPCs and MM cells according to Amazonia! Atlas. **B** qRT-PCR analysis of ALKBH5 expression in normal peripheral blood mononuclear cells (PBMCs) from healthy donors (black), primary cells derived from patients with MM (red), and human myeloma cell lines (blue), using GAPDH as the reference. **C** ALKBH5 expression in samples from healthy donors (HD) or patients diagnosed with monoclonal gammopathy of undetermined significance (MGUS) and smoldering MM (SMM) in Zhan et al. [29] myeloma dataset according to Oncomine database. **D** ALKBH5 expression in samples from patients diagnosed with MM and PCL in the dataset GSE39925 [30]. **E** Normalized expression signal of ALKBH5 in various hematological malignancies according to the Amazonia! Atlas. CLL Chronic lymphocytic leukemia; T-ALL T-cell acute lymphoblastic leukemia; B-ALL B-cell acute lymphoblastic leukemia; CML Chronic myeloid leukemia; AML Acute myeloid leukemia; B-NHL B-cell non-Hodgkin lymphoma; T-NHL T-cell non-Hodgkin lymphoma; MDS Myelodysplastic syndromes. **F** *ALKBH5* mRNA expression in MM and other indicated cancer cell lines, according to the Cancer Cell Line Encyclopedia dataset. T-cell ALL T-cell acute lymphoblastic leukemia; DLBCL Diffuse large B-cell lymphoma; B-cell ALL B-cell acute lymphoblastic leukemia; lung NSC Non-small cell lung cancer; AML Acute myeloid leukemia; CML Chronic myeloid leukemia. **G, H** Kaplan–Meier survival analysis in indicated myeloma datasets [31, 32]. Patients were divided into two groups based on ALKBH5 expression levels (high and low). Cutoff values were determined by the maximum standardized log-rank statistic. *P*-value was calculated by the log-rank test. ^*^P < 0.05, ^**^P < 0.01, ^***^P < 0.001, and ^****^P < 0.0001 (*t*-test). ns No significance. Error bars denote mean ± SD.

The publicly available MM datasets were queried to examine the expression profile of m^6^A modifiers in MM. The Amazonia! (amazonia.transcriptome.eu) MM atlas showed that the expression of m^6^A demethylase ALKBH5, but not of other m^6^A modifiers (METTL3, METTL14, WTAP, or FTO), was significantly higher in MM cells compared with the normal BM plasma cells (nBMPCs) (Figs. 1A and S1A). Quantitative PCR (qPCR) analyses also showed that *ALKBH5* mRNA levels were significantly higher in purified primary myeloma cells and established human myeloma cell lines (HMCLs) compared with peripheral blood mononuclear cells (PBMCs) from healthy donors (Fig. 1B). Gene Expression Omnibus (GEO) dataset analysis demonstrated that the elevated ALKBH5 expression was correlated with myeloma disease progression from normal to smoldering myeloma (SMM) (Fig. 1C) [29] and from MM to plasma cell leukemia (PCL) (Fig. 1D) [30]. Moreover, according to Amazonia! Atlas, ALKBH5 expression was significantly higher in MM compared with other hematological malignancies (Fig. 1E). The Cancer Cell Line Encyclopedia (CCLE) dataset also revealed that ALKBH5 was highly expressed in MM compared with other cancer cell lines (Fig. 1F). In addition, we observed that the top-ranked tumors with high ALKBH5 expression were largely lymphocyte malignancies (Fig. 1E, F).

Next, we investigated the prognostic value of these five canonical m^6^A modifiers in MM. According to the Arkansas myeloma cohort (GEO accession number GSE4581) [31], the elevated expression of two m^6^A demethylases (ALKBH5 and FTO) was correlated with poor patient survival (Figs. 1G and S1E). Conversely, the high expression of WTAP predicted improved patient survival, while the expression of METTL3 and METTL14 was not significantly associated with prognosis in MM (Fig. S1B–D). We also validated that patients with high ALKBH5 or FTO expression had significantly shorter overall survival compared with those with low expression in Mulligan et al. [32] myeloma cohort (GSE9782) (Figs. 1H and S1F). Next, we knocked down all the five m^6^A modifiers in HMCLs using short hairpin RNAs (shRNAs) to clarify their functions in MM (Fig. S1G). Similar to the results of survival analysis (Figs. 1G, H; S1B–F), the knockdown (KD) of *ALKBH5* or *FTO* inhibited the proliferation of HMCLs (Fig. S1H). These phenomena indicated that the hypomethylation of mRNAs mediated by m^6^A erasers (ALKBH5 and FTO) could promote tumorigenesis in MM. Therefore, we compared the expression pattern of FTO with that of ALKBH5 in MM. We also found that FTO was not significantly overexpressed in MM cells based on another MM atlas [33] and our own samples (Fig. S1I, J). Unlike ALKBH5, FTO expression in MM was not high compared with other blood cancers and solid tumors (Fig. S1K, L). Collectively, increased ALKBH5 expression was correlated with a poor prognosis of MM. Thus, we focused on exploring the role of ALKBH5 in myeloma tumorigenesis.

### ALKBH5 played a pro-proliferative and -survival role in MM cells in vitro

**Fig. 2:**
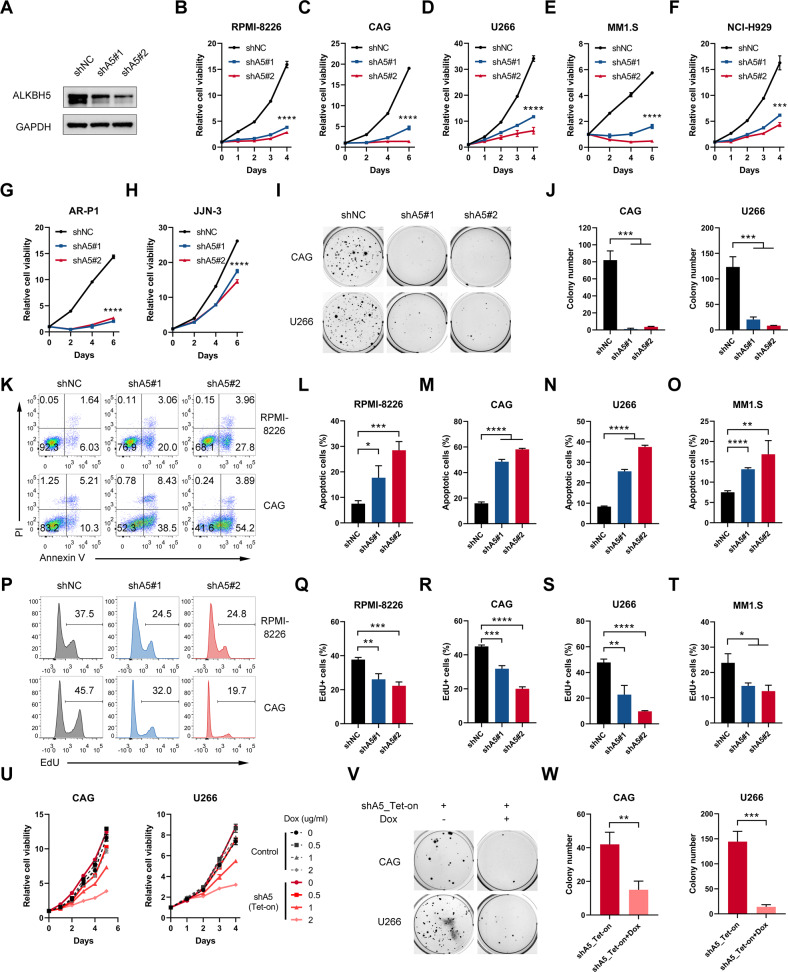
ALKBH5 played a pro-proliferative and -survival role in MM cells in vitro. **A** Immunoblotting of ALKBH5 after shRNA-mediated KD in RPMI-8226 cells. **B** Proliferation of MM cells RPMI-8226, **C** CAG, **D** U266, **E** MM1.S, **F** NCI-H929, **G** AR-P1, **H** JJN-3 transduced with indicated lentiviruses, as determined by CCK-8. **I, J** Representative colony images (**I**) and statistics of colony counts (**J**) of CAG and U266 cells after stable transduction of the indicated lentiviruses. **K**–**O** Apoptotic analysis of MM cells with shNC or shALKBH5s. Representative flow cytometry plots from RPMI-8226 and CAG cells (**K**) and statistics of the percentage of apoptotic (Annexin V + ) cells in RPMI-8226 (**L**), **M** CAG, **N** U266, and MM1.S (**O**) cells transduced with indicated lentiviruses. **P**–**T** EdU assay of MM cells with shNC or shALKBH5s. Representative flow cytometry plots from RPMI-8226 and CAG cells (**P**) and statistics of the percentage of EdU-positive cells in RPMI-8226 (**Q**), **R** CAG, **S** U266, **T** MM1.S. **U** Growth curves of MM cells after transduction with control or shALKBH5_Tet-on lentiviruses and treatment with Dox at indicated concentrations. **V, W** Representative colony images (**V**) and statistics of colony number (**W**) of CAG and U266 cells after ALKBH5 KD by Dox (1 μg/mL). ^*^P < 0.05, ^**^P < 0.01, ^***^P < 0.001, and ^****^P < 0.0001 (*t*-test). ns No significance. Error bars denote mean ± SD.

Both gain- and loss-of-function studies were performed to investigate the role of ALKBH5 in MM. We conducted shRNA-mediated KD experiments (shA5#1 and shA5#2) to decrease ALKBH5 expression in HMCLs (Fig. 2A). Consequently, the depletion of ALKBH5 had a significant inhibitory effect on cellular growth (Fig. 2B–H), and colony formation (Fig. 2I, J) compared with that in the control group. ALKBH5 KD significantly induced cell apoptosis (Fig. 2K–O) and decreased DNA synthesis (Fig. 2P–T) compared with that in the control group, as revealed by apoptosis and 5-ethynyl-2'-deoxyuridine (EdU) assays, respectively. The cell cycle analysis showed that ALKBH5 KD in MM cells increased the proportion of cells in the G0/G1 phase and decreased the proportions of cells in the S and G2/M phases (Fig. S2A, B). In contrast, the overexpression of ALKBH5 (Fig. S2C) significantly promoted the growth of HMCLs (Fig. S2D–H). Furthermore, we established doxycycline (Dox)-inducible ALKBH5 conditional KD MM cell lines (shA5_Tet-on), and conditional depletion of ALKBH5 (Fig. S2I) significantly inhibited MM cell proliferation (Fig. 2U) and colony formation (Fig. 2V, W). Together, these results indicated that ALKBH5 plays a major role in the proliferation of human MM cells in vitro.

### ALKBH5 promoted MM cell growth in vivo

**Fig. 3:**
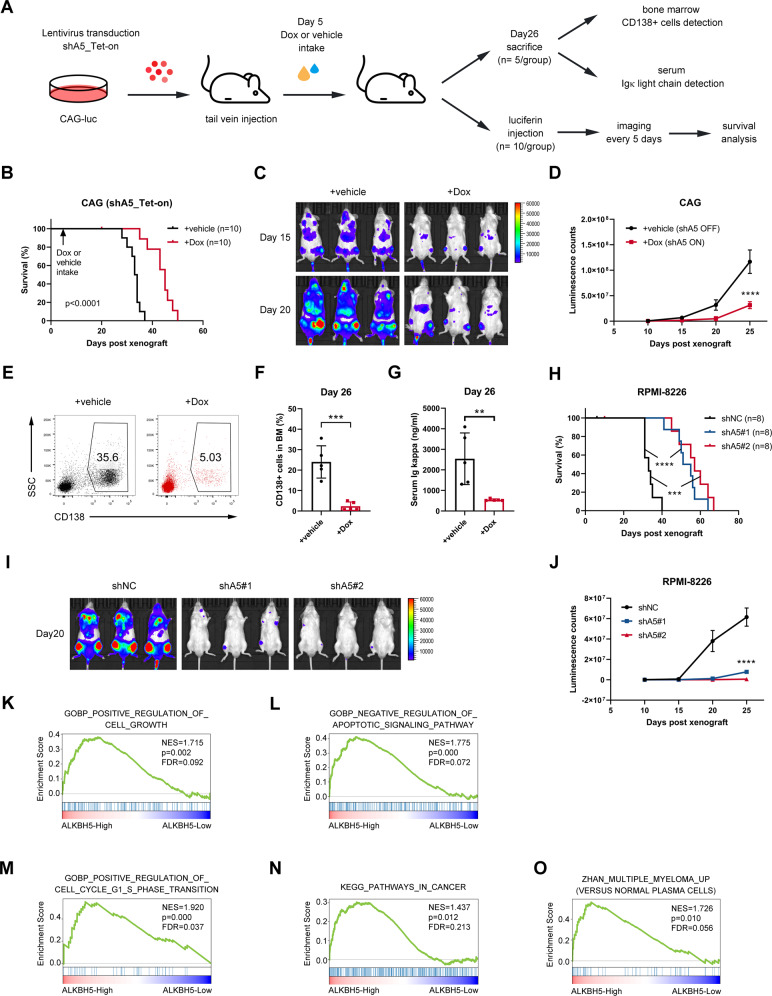
ALKBH5 promoted MM cell growth in vivo. **A** Experimental scheme for (**B**–**G**). **B** Kaplan–Meier survival curve of NCG mice xenografted with the luciferase-labeled CAG cells transduced with Dox-inducible shALKBH5 (shA5_Tet-on) subjected to the vehicle or Dox treatment (*n* = 10/group). **C, D** Representative images of chemiluminescence (**C**) and summary measurement of signals (**D**). **E, F** Representative flow plot (**E**) and statistics of the percentage of human CD138-positive cells in BM samples prepared 26 days after xenograft from the vehicle and Dox groups (*n* = 5/group). **G** Concentration of Igκ light chain detected in serum from xenografted mice using ELISA, either mock-treated (vehicle) or with ALKBH5 KD (Dox) (*n* = 5/group). **H**–**J** Kaplan–Meier survival curve (**H**), representative image (20 days post-xenograft) (**I**), and summary of luminescence signals (**J**) of mice implanted with luciferase-labeled RPMI-8226 cells with or without stable KD of ALKBH5 (*n* = 8/group). **K**–**O** GSEA plots show that high ALKBH5 expression is positively related to gene sets related to positive regulation of cell growth, **K** Negative regulation of apoptotic signaling pathway, **L** Positive regulation of cell cycle G1 and S phase transition, **M** pathways in cancer, **N** and Zhan multiple myeloma up (**O**) in 304 MM patients based on a transcriptome data set (GSE26760 [34]). The cohort was divided into ALKBH5-High (top 50%) and ALKBH5-Low (bottom 50%) groups. ^*^P < 0.05, ^**^P < 0.01, ^***^P < 0.001, and ^****^P < 0.0001 (*t*-test). ns, No significance. Error bars denote mean ± SD.

We generated various xenograft models via tail vein injection of the luciferase-labeled MM cell lines into immune-deficient NOD CRISPR *Prkdc Il2r* *Gamma* (NCG) mice to evaluate the role of ALKBH5 in MM cell growth in vivo (Figs. 3A–J and S3A–C). In the first xenograft assay, ALKBH5 KD of CAG cells was achieved using Dox-inducible shRNA (Fig. 3A). Chemiluminescence imaging (Fig. 3C, D) and detection of MM cells in BM (Fig. 3E, F) revealed that the bone infiltration of MM cells and subsequent malignant expansion were significantly suppressed by ALKBH5 KD induced by Dox intake (Dox *vs*. vehicle). As a result, shALKBH5-ON (Dox) group displayed significantly alleviated tumor burden, as revealed by the concentration of immunoglobulin kappa (Igκ) light chain in serum (Fig. 3G) and prolonged survival (Fig. 3B) compared with the shALKBH5-OFF (vehicle) group. We also generated xenograft models using MM cell lines with stable ALKBH5 KD (Fig. 3H–J) or ALKBH5 overexpression (Fig. S3A–C). As expected, shALKBH5 significantly delayed human MM cell RPMI-8226 progression and prolonged survival in recipient mice compared with shNC (Fig. 3H–J). Conversely, the overexpression of ALKBH5 significantly increased the tumorigenic potential of MM cell line U266 and had an adverse impact on the survival in xenografted models (Fig. S3A–C). Furthermore, gene set enrichment analysis (GSEA) with transcriptome datasets of primary MM samples (GSE26760 [34] and GSE19784 [35]) showed that high ALKBH5 expression was positively correlated to genes related to cell growth, anti-apoptosis signaling, cell cycle progression, cancer pathways, and genes upregulated in MM (Figs. 3K–O and S3D–M). Taken together, our MM cell xenograft models and GSEA of myeloma patient data sets supported the role of ALKBH5 in the malignant progression of myeloma in vivo.

### Demethylation activity was required for ALKBH5 to promote MM cell growth

**Fig. 4:**
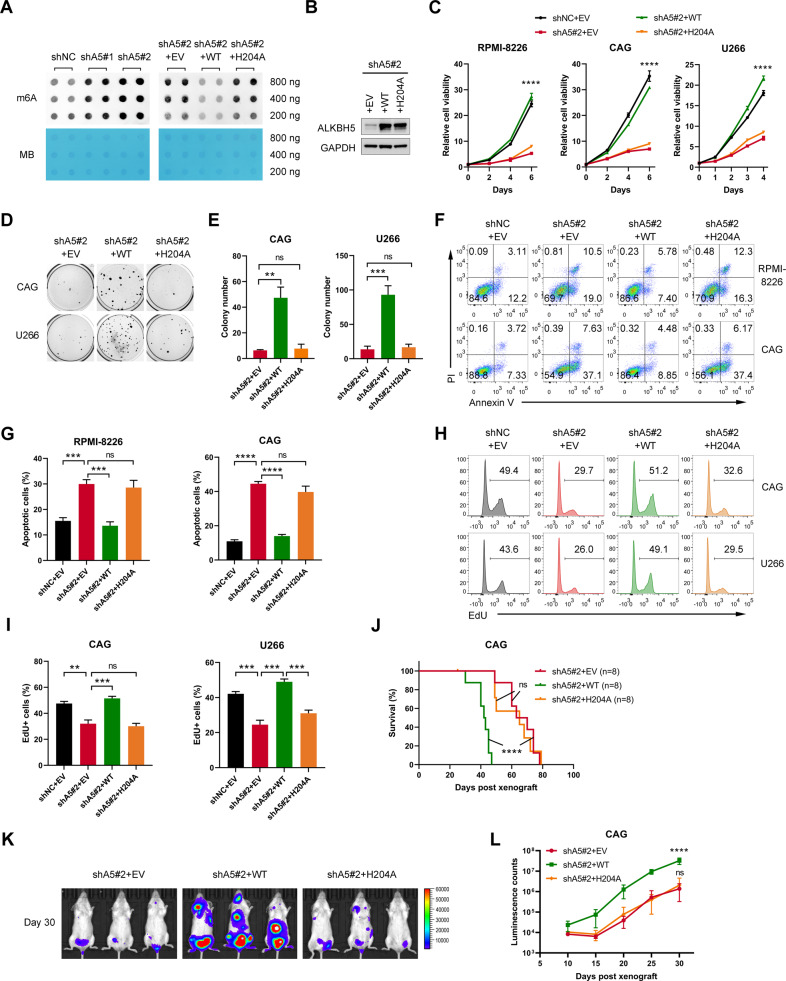
ALKBH5 promoted MM growth in an m^6^A-dependent manner. **A** m^6^A dot blot assays of global m^6^A abundance in CAG cells transduced with indicated lentiviruses (*n* = 2 biological replicates). Methylene blue staining (MB) was used as a loading control. **B** Western blots of ALKBH5 in MM cells transduced with indicated lentiviruses. GAPDH was used as a loading control. **C** Growth curves of MM cells transduced with indicated lentiviruses. **D, E** Representative colony images (**D**) and statistics of colony number (**E**) of MM cells after the transduction of the indicated lentiviruses. **F, G** Representative flow plots (**F**) and statistics of the percentage of apoptotic cells (**G**) in RPMI-8226 and CAG cells transduced with indicated lentiviruses. **H, I** Representative flow plots (**H**) and statistics of the percentage of EdU-positive cells (**I**) in CAG and U266 cells transduced with indicated lentiviruses. **J**–**L** Kaplan–Meier curve for the survival (**J**), representative image (**K**; 30 days post-xenograft), and statistics of luminescence counts (**L**) of mice xenografted with luciferase-labeled CAG cells that transduced with indicated lentiviruses (*n* = 8/group). ^*^P < 0.05, ^**^P < 0.01, ^***^P < 0.001, and ^****^P < 0.0001 (*t*-test). ns, No significance. Error bars denote mean ± SD.

It was determined whether demethylation activity was required for ALKBH5 function. First, we found that global m^6^A abundance increased as a result of ALKBH5 KD in MM cells, as assessed by the dot blot assay (Fig. 4A, left panel), which confirmed the m^6^A demethylation activity of ALKBH5 in MM cells. Next, we restored ALKBH5 expression by forced expression of wild-type (WT) ALKBH5 or ALKBH5-H204 A mutant (a catalytically inactive mutant [12]) (Fig. 4B). The dot blot assay showed that the WT-ALKBH5, but not the H204A mutant, restored the global m^6^A level of ALKBH5 KD cells (Fig. 4A, right panel). Consecutively, we observed that the restoration of WT-ALKBH5 substantially rescued the proliferation (Fig. 4C) and colony formation of ALKBH5-KD HMCLs (Fig. 4D, E). However, the ALKBH5 H204A mutant failed to rescue cell growth inhibition caused by ALKBH5 deficiency (Fig. 4C–E). Similarly, the biological changes, including apoptosis induction (Fig. 4F, G) and DNA synthesis disruption (Fig. 4H, I) caused by ALKBH5 KD in MM cells, were rescued by WT-ALKBH5 but not H204A ALKBH5. Moreover, the disruption of tumor formation by shALKBH5 was rescued by the overexpression of the WT-ALKBH5 but not the catalytic inactive mutant in vivo (Fig. 4J–L). These results confirmed that intact demethylase activity was required for the oncogenic function of ALKBH5 in MM.

### Identification of potential downstream targets of ALKBH5 in MM

**Fig. 5:**
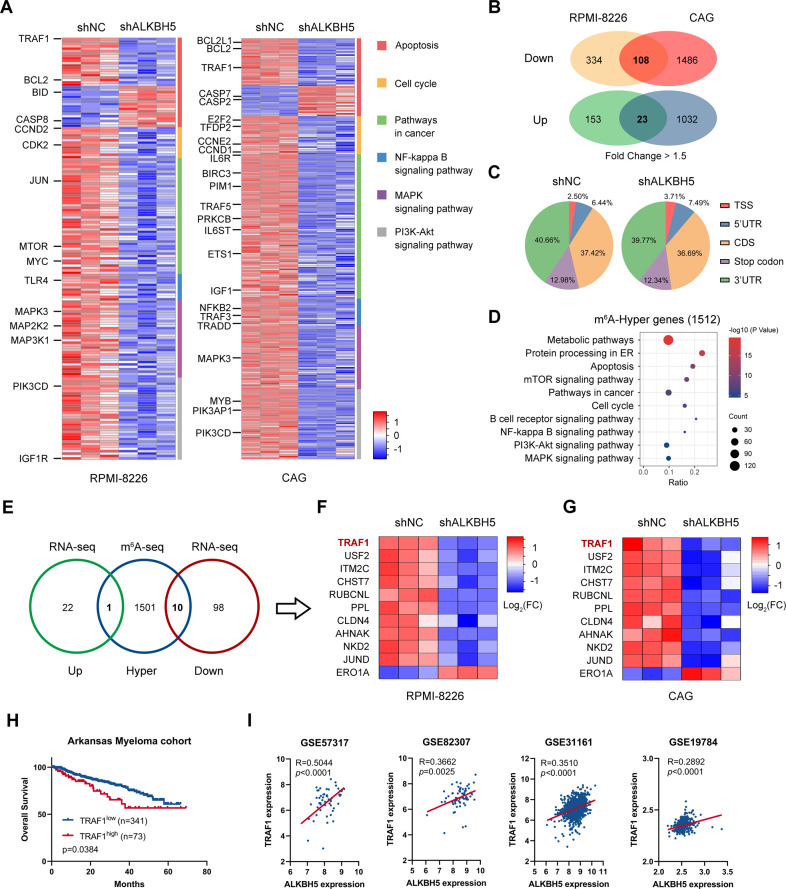
Identification of potential downstream targets of ALKBH5 in MM cells. **A, B** RNA-seq analysis of MM cells upon ALKBH5 KD. Heatmap (**A**) shows differential expression of indicated KEGG pathway genes in RPMI-8226 (left panel) and CAG (right panel) upon ALKBH5 KD. Venn diagram (**B**) shows the number of genes with significant changes in expression (fold change > 1.5) upon ALKBH5 KD in RPMI-8226 and CAG cells. **C, D** m^6^A-seq analysis of ALKBH5 KD in RPMI-8226 cells. Distribution of total m^6^A peaks in the indicated regions of mRNA transcripts in the control and ALKBH5 KD cells (**C**). Bubble plot (**D**) shows KEGG pathway analysis of the genes with significantly increased m^6^A abundance in ALKBH5 KD cells (*P* < 0.05). **E** Integrative analysis of RNA-seq and m^6^A-seq to identify the potential targets of ALKBH5 in MM. Up or Down indicates genes with significantly increased or decreased expression upon ALKBH5 KD in both RPMI-8226 and CAG cells as detected by RNA-seq (fold change > 1.5), respectively. Hyper indicates genes with significantly higher m^6^A abundance in m^6^A-Seq (*P* < 0.05). **F, G** Heatmap shows differential expression of overlapped genes in RMPI-8226 (**F**) and CAG (**G**) upon ALKBH5 KD. **H** Kaplan–Meier survival analysis in the Arkansas myeloma dataset [31]. The cutoff value was determined by the maximum standardized log-rank statistic. *P*-value was detected using the log-rank test. **I** Pearson’s correlation between *ALKBH5* and *TRAF1* mRNA expression in the indicated datasets [35–38]. The expression values were log_2_ transformed.

RNA sequencing (RNA-seq) was conducted to compare the gene expression profile of MM cells with or without ALKBH5 KD to explore the potential mechanisms underlying ALKBH5 function in MM. A total of 2896 and 4570 genes were differentially expressed in cell lines RPMI-8226 and CAG, respectively (Fig. S4A, B). Several of these differentially expressed genes were enriched in apoptosis, cell cycle, pathways in cancer, nuclear factor-kappa B (NF-κB) signaling pathway, mitogen-activated protein kinase (MAPK) signaling pathway, and phosphatidylinositol 3-kinase (PI3K)-Akt signaling pathway, according to the Kyoto Encyclopedia of Genes and Genomes (KEGG) pathway enrichment analysis (Fig. 5A), which was consistent with the phenotype alterations caused by ALKBH5 KD and GSEA of patient datasets. The differentially expressed genes by at least 1.5-fold in both cell lines were overlapped as the candidates of direct targets of ALKBH5 (Fig. 5B). Then, we compared the m^6^A methylomes in shNC and shALKBH5 RPMI-8226 cells by m^6^A sequencing (m^6^A-seq). Consistent with previous reports [8, 9], our m^6^A-seq data showed that m^6^A peaks were mainly distributed in the coding region (CDS) and 3'-untranslated region (3'-UTR) of transcripts in MM cells, displaying the consensus motif DRACH (Fig. 5C, Fig. S4C, D). Given the demethylase activity of ALKBH5, up to 1512 genes with significantly elevated m6A peaks upon ALKBH5 KD were identified for KEGG pathway enrichment (Figs. 5D and S4E). Also, the proliferation-associated pathways detected by RNA-seq were significantly enriched in genes with m^6^A-hyper peaks (Fig. 5D).

Integrative analysis of the RNA-seq and m^6^A-seq data identified 10 downregulated and 1 upregulated potential targets of ALKBH5 in MM (Fig. 5E–G). The qPCR data validated that most of these genes were significantly regulated by ALKBH5 in accordance with RNA-seq results (Fig. S5A, B). Moreover, the prognostic value of the 11 genes in MM was investigated. We found that high expression of TRAF1, CHST7 (excluded by qPCR data), PPL, and ERO1A (excluded by qPCR data and unexpected prognostic impact) was correlated with poor survival of MM patients in the Arkansas myeloma cohort [31], whereas the other potential targets of ALKBH5 did not show a prognostic significance in MM (Figs. 5H; S5C–L). Furthermore, gene expression analysis by querying GEO datasets (GSE57317 [36], GSE82307 [37], GSE31161 [38], and GSE19784 [35]) validated the significant positive correlation between ALKBH5 and TRAF1 expression in samples from MM patients (Fig. 5I) but not between ALKBH5 and PPL expression (Fig. S5M and N).

Hence, TRAF1 may be responsible for the altered phenotype following ALKBH5 intervention in MM cells. Tumor necrosis factor receptor (TNFR)-associated factor 1 (TRAF1) is an adapter molecule known for its role in TNFR-induced cell survival [39]. In normal conditions, TRAF1 expression was largely restricted to activated lymphocytes, dendritic cells, and certain epithelia [40, 41]. Several lymphoid malignancies, especially B-cell types, displayed high levels of TRAF1 expression [39, 41–46]. Besides, TRAF1 overexpressed and played a critical oncogenic role in other types of cancers [47–49]. We found that TRAF1 was highly expressed in MM and shared a similar expression pattern with ALKBH5 (Fig. S5O–R). Thus, we focused on TRAF1 as a potential key target of ALKBH5 in subsequent studies.

### TRAF1 was a direct target of ALKBH5 in MM

**Fig. 6:**
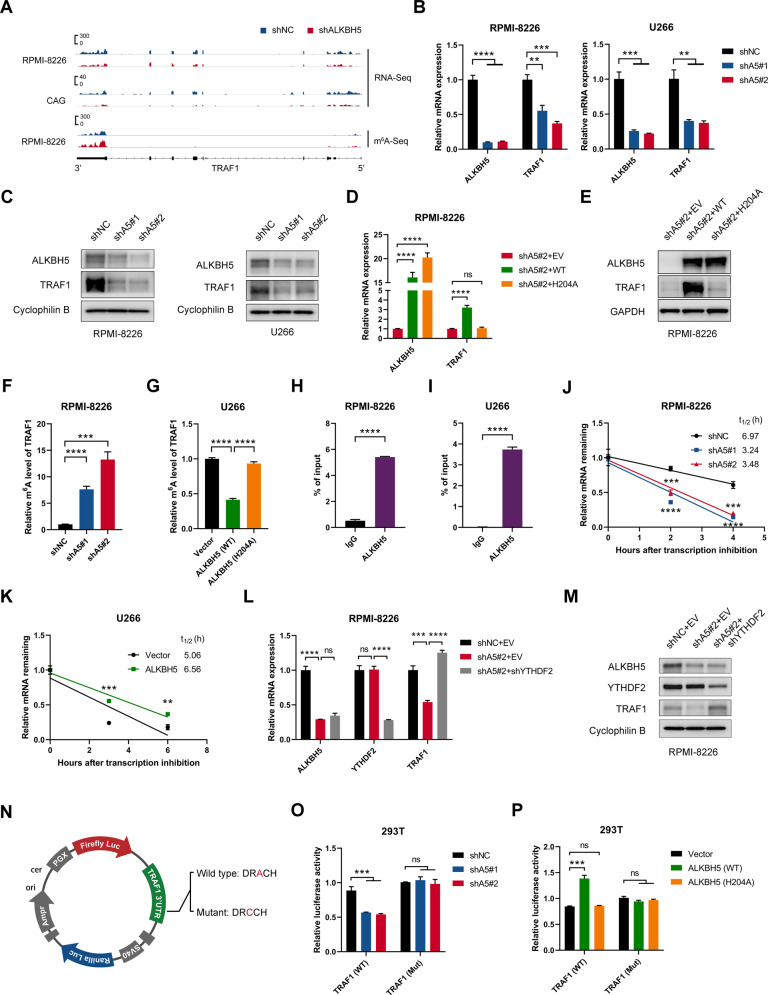
ALKBH5 regulated TRAF1 expression by altering its mRNA stability in an m^6^A-dependent manner. **A** IGV tracks displaying the mRNA (top) and m^6^A (bottom) abundance in TRAF1 transcripts in control and ALKBH5 KD MM cells as detected by RNA-seq and m^6^A-seq. **B** qRT-PCR analysis showing TRAF1 levels in MM cells transduced with indicated lentiviruses. **C** Western blots of ALKBH5 and TRAF1 in MM cells transduced with indicated lentiviruses. Cyclophilin B was used as a loading control. **D, E** Relative mRNA level (**D**) and Western blots (**E**) of ALKBH5 and TRAF1 in MM cells transduced with indicated lentiviruses. GAPDH was used as a loading control. **F** m^6^A-IP-qPCR analysis of m^6^A enrichment on *TRAF1* mRNA in RPMI-8226 cells with or without ALKBH5 KD. **G** m^6^A-IP-qPCR analysis of m^6^A enrichment on *TRAF1* mRNA in U266 cells transduced with indicated lentiviruses. **H, I** ALKBH5-RIP-qPCR validation of ALKBH5 binding to *TRAF1* mRNA in RPMI-8226 (**H**) and U266 (**I**) cells. **J, K** mRNA half-life (t_1/2_) of *TRAF1* in RPMI-8226 (**H**) and U266 (**I**) cells transduced with indicated lentiviruses. **L** qRT-PCR analysis of *TRAF1* mRNA expression in MM cells transduced with indicated lentiviruses. **M** Immunoblotting for ALKBH5, YTHDF2, and TRAF1 in RPMI-8226 cells after transduction with indicated lentiviruses. Cyclophilin B was used as a loading control. **N** Graphical representation of dual-luciferase reporters. The wild-type or mutant (m^6^A motif DRACH mutated) sequence of TRAF1–3'-UTR was cloned into pmirGLO vector between a firefly and Renilla luciferase elements. **O, P** Relative luciferase activity of the wild-type or mutant TRAF1–3'-UTR dual-luciferase reporter in 293 T cells treated with indicated lentivirus. Relative luciferase activity was calculated using the ratio of firefly and Renilla luciferase activity values. ^*^P < 0.05, ^**^P < 0.01, ^***^P < 0.001, and ^****^P < 0.0001 (*t*-test). ns No significance. Error bars denote mean ± SD.

In line with RNA-seq data (Fig. 6A), our qPCR results confirmed the decreased mRNA level of *TRAF1* following ALKBH5 KD in MM cells (Figs. 6B and S6A). ALKBH5 KD also decreased the TRAF1 protein level in MM cell lines (Fig. 6C). On the other hand, the forced expression of the wild-type ALKBH5, but not the mutant H204A, restored TRAF1 expression in ALKBH5 KD cells (Figs. 6D, E; S6B, C). These findings indicated that ALKBH5 regulated TRAF1 expression through its demethylation activity. In addition, we found that TRAF1 expression was significantly upregulated by the KD of m^6^A methyltransferase METTL3 (Fig. S6F, G), which confirmed that TRAF1 was regulated by m^6^A modulators. Next, we performed m^6^A immunoprecipitation (m^6^A-IP), followed by TRAF1-specific qPCR to substantiate the change in TRAF1 m^6^A methylation levels following ALKBH5 KD. Consistent with the current m^6^A-seq results (Fig. 6A), TRAF1 transcripts showed an increased m^6^A level after ALKBH5 KD (Fig. 6F and S6H). Conversely, the forced expression of WT-ALKBH5, but not the H204A mutant ALKBH5, decreased the m^6^A abundance of *TRAF1* mRNA in MM cells (Fig. 6G). Furthermore, we conducted RNA immunoprecipitation (RIP) of ALKBH5 in MM cells and found that TRAF1 transcripts were significantly enriched, suggesting that ALKBH5 is bound to TRAF1 transcripts directly (Figs. 6H, I; S6I). Together, these results indicated that TRAF1 was a direct downstream target of ALKBH5 in MM.

### ALKBH5 regulated *TRAF1* mRNA stability by altering m^6^A modification

RNA m^6^A modification was reported to affect mRNA stability [14–16]. The loss of mRNA stability could explain the reduced *TRAF1* mRNA expression in ALKBH5 KD cells. Therefore, we conducted an RNA decay assay using the transcription inhibitor actinomycin D to assess the change of *TRAF1* mRNA stability following ALKBH5 KD or overexpression in MM cells. Consequently, ALKBH5 KD significantly decreased *TRAF1* mRNA half-life (Fig. 6J), while the overexpression of ALKBH5 prolonged the half-life of TRAF1 transcripts (Fig. 6K). The various biological effects of mRNA m^6^A modification are effectuated by different m^6^A readers [50]. YTH domain-containing family protein 2 (YTHDF2) is the major m^6^A reader responsible for the degradation of its target m^6^A modified transcripts [14, 15]. Thus, we knocked down *YTHDF2* in ALKBH5 KD MM cells and observed that TRAF1 expression was restored (Figs. 6L, M; S6J), suggesting that YTHDF2 mediated the effect of ALKBH5 on *TRAF1* mRNA. In m^6^A-seq, ALKBH5 deficiency markedly increased m^6^A abundance in the 3'-UTR of TRAF1 transcripts (Fig. 6A). We performed the TRAF1 3'-UTR-reporter dual-luciferase assay to determine the role of TRAF1 3'-UTR (Fig. 6N) and found that the activity of luciferase decreased upon ALKBH5 KD, while mutations at m^6^A motif sites (A to C) in TRAF1 3'-UTR resulted in resistance to the effect of ALKBH5 KD (Fig. 6O). In contrast, the forced expression of WT-ALKBH5, but not H204A mutant, increased the activity of luciferase, while the mutation of m^6^A sites also abolished this effect (Fig. 6P). Together, these findings suggested that ALKBH5 regulated YTHDF2-mediated *TRAF1* mRNA stability in MM cells via m^6^A modifications in the 3'-UTR of TRAF1 transcript.

### TRAF1 was a major contributor to the function of ALKBH5 in MM

**Fig. 7:**
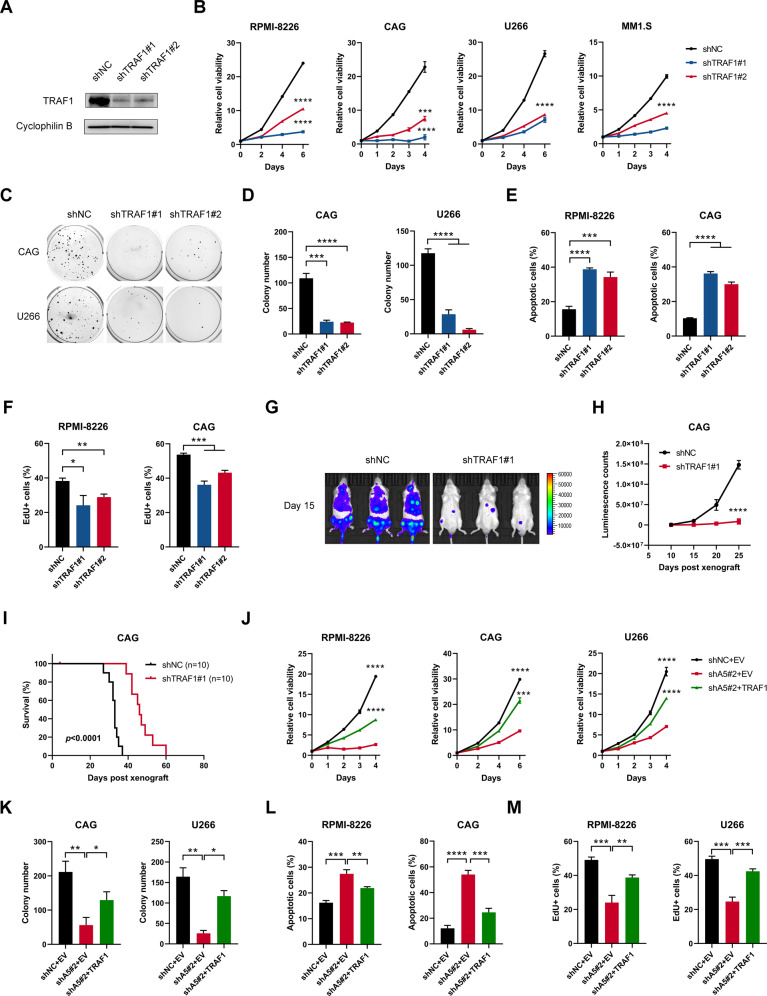
TRAF1 was a functionally important target of ALKBH5 in MM. **A** Western blots show the KD efficiency of TRAF1 in MM cells. **B** Effects of TRAF1 KD on cell growth in MM cells. **C, D** Representative colony images (**C**) and statistics of colony counts (**D**) of CAG and U266 cells after TRAF1 KD. **E** Statistics of the percentage of apoptotic cells of MM cells upon TRAF1 KD (see also Fig. S7A). **F** Statistics of the percentage of EdU-positive cells in RPMI-8226 and CAG cells upon TRAF1 KD (see also Fig. S7B). **G**–**I** Representative image (**G**; 15 days post-xenograft), a summary of luminescence counts (**H**), and Kaplan–Meier curve for the survival (**I**) of mice implanted with luciferase-labeled CAG cells with or without TRAF1 KD (*n* = 8/group). **J**–**M** Effects of TRAF1 restoration on cell proliferation (**J**), colony-forming capacity (**K**), apoptosis (**L**), and DNA synthesis (revealed by EdU; **M**) of ALKBH5 KD MM cells (see also supplemental S7L–N). ^*^P < 0.05, ^**^P < 0.01, ^***^P < 0.001, and ^****^P < 0.0001 (*t*-test). ns No significance. Error bars denote mean ± SD.

Since TRAF1 could be a direct target of ALKBH5, we sought to study the function of TRAF1 in MM. In line with the phenotype resulting from ALKBH5 KD, TRAF1 KD significantly inhibited cell growth and colony formation in HMCLs (Fig. 7A–D). Moreover, TRAF1 KD induced apoptosis and inhibited DNA duplication in MM cells (Figs. 7E, F; S7A, B), which mimicked the effects of ALKBH5 depletion. Cell cycle analysis of MM cells post-KD of TRAF1 also detected defects in the G1-S phase transition (Fig. S7C, D). In addition, our in vivo study also confirmed that TRAF1 KD substantially reduced MM tumor formation in xenograft mice (Fig. 7G–I). On the other hand, the in vitro study showed that the overexpression of TRAF1 promoted MM growth (Fig. S7E–J). Thus, it could be speculated that TRAF1 mediated the function of ALBKH5 in MM cells. Next, we restored TRAF1 expression in ALKBH5 KD MM cells to clarify whether TRAF1 was a significant contributor to the function of ALKBH5 in MM proliferation (Fig. S7K) and found that the growth inhibitory effects caused by ALKBH5 deficiency were largely rescued by restoration of TRAF1 expression (Figs. 7J–M; S7L–N). Overall, these data suggested that TRAF1 was a functional target of ALKBH5 in MM.

### ALKBH5-TRAF1 regulated NF-κB and MAPK pathways in MM

**Fig. 8:**
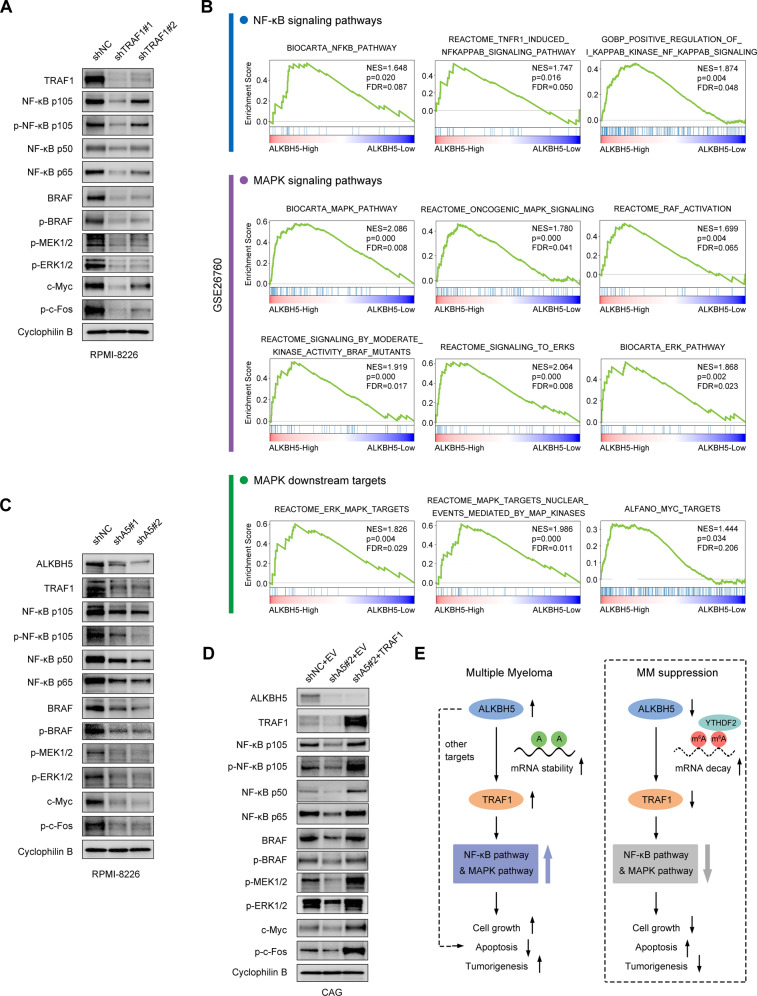
ALKBH5-TRAF1 regulated NF-κB and MAPK pathways in MM. **A** Western blots of TRAF1, NF-κB p105, P-NF-κB p105 (Ser932), NF-κB p50, NF-κB p65, BRAF, p-BRAF (Ser445), p-MEK1/2 (Ser221), p-ERK1/2 (Thr202/Tyr204), c-Myc, and p-c-Fos (Ser32) in RPMI-8226 cells with or without TRAF1 KD. Cyclophilin B was used as a loading control. **B** GSEA plots show enriched gene sets of NF-κB and MAPK signaling pathways and MAPK downstream targets in ALKBH5-High (top 50%) *vs*. ALKBH5-Low (bottom 50%) groups in a cohort of patients with MM (GSE26760 [34]). **C, D** Western blots of NF-κB and MAPK pathway molecules in MM cells upon ALKBH5 KD (**C**) and TRAF1 restoration (**D**). Cyclophilin B was used as a loading control. **E** Proposed model showing the role of ALKBH5 in MM pathogenesis.

Since ALKBH5 upregulates TRAF1 expression and contributes to MM growth, we sought to explore the specific mechanism underlying their oncogenic actions. TRAF1 contributes to apoptosis resistance in the TNFR signaling pathway as part of the complex with TRAF2, where it promotes NF-κB activation through recruitment of cellular inhibitor of apoptosis protein (cIAP) [39, 51]. Reportedly, TRAF1 enhances the MAPK pathway by affecting TRAF2-mediated Lys48-linked ubiquitination of the serine/threonine-protein kinase B-raf (BRAF) [48]. TRAF1 KD led to decreased BRAF protein expression, decreased downstream MEK/ERK activation, and inhibited cell growth in human lung cancer cell lines [48]. Consistently, we found that TRAF1 inhibition also downregulated NF-κB and MAPK pathways in MM cells (Fig. 8A). Interestingly, we observed significant enrichment of the gene sets of NF-κB and MAPK pathways in ALKBH5 high-expression cases from the cohorts of patients with MM (GSE26760 [34] and GSE19784 [35]) by GSEA (Figs. 8B; S8A, B), suggesting that ALKBH5 also positively regulated these pathways. Thus, we proposed that ALKBH5 enhances TRAF1-mediated activation of NF-κB and MAPK pathways in MM cells, followed by the promotion of cell growth. As expected, knocking down ALKBH5 in MM cells not only led to TRAF1 suppression but also reduced the activation of downstream NF-κB and MAPK signaling molecules (Fig. 8C), which was rescued by TRAF1 restoration (Fig. 8D). Collectively, these data indicated that the ALKBH5/m^6^A/TRAF1 axis regulated MAPK and NF-κB pathways to promote survival and cell growth in MM cells (Fig. 8E).

### ALKBH5 expression in MM subgroups with various genetic features

We analyzed ALKBH5 expression in several MM cohorts containing different genetic subgroups, most of which were defined via immunoglobulin heavy-chain locus (IgH; at 14q32) translocations and cyclin D overexpression (TC classification) [31, 52], to determine whether ALKBH5 overexpression was associated with recurrent genetic changes in MM. ALKBH5 expression levels did not show a significant difference between patients with TC populations and unclassified group (None) in Mulligan et al. [32] and HOVON65/GMMG-HD4 [35] cohort (Fig. S9A, B). Interestingly, ALKBH5 expression was significantly lower in the hyperdiploid group compared with other clusters with IgH translocations in both HOVON65/GMMG-HD4 [35] and Zhan et al. [31] cohorts (Fig. S9B, C). Previous studies suggested that hyperdiploidy conferred a relatively favorable prognosis in MM [53–56], which was consistent with our finding that lower ALKBH5 expression predicted a better prognosis. Moreover, in line with the activation of the NF-κB pathway by ALKBH5, we also observed high ALKBH5 expression in a subgroup (NFκB) characterized by the high expression of genes involved in the NF-κB pathway in the HOVON65/GMMG-HD4 [35] cohort (Fig. S9B). In addition, ALKBH5 was highly expressed in the MMSET group in all cohorts (Fig. S9A–C), indicating that ALKBH5 might be regulated by dimethylation of histone H3 at lysine 36 (H3K36me2) mediated by histone methyltransferase MMSET [3]. However, ALKBH5 expression was not altered significantly upon MMSET KD nor alterations of MMSET translocation in MM cell line KMS11 according to GEO datasets (GSE29147 [57], GSE29148 [57], GSE57863 [58], and GSE24746 [3]) (Fig. S9D–G). Furthermore, compared with patients with no 1q21 amplification (two copies), patients with three copies showed a significant increase in ALKBH5 expression in the cohort by Zhan et al. [31] (Fig. S9H). However, ALKBH5 expression did not show a significant difference between two copies and more than four copies (Fig. S9H). Overall, MM with IgH translocations showed higher ALKBH5 expression compared with the hyperdiploid group, and ALKBH5 expression was not likely to be regulated by MMSET in MM cells.

## Discussion

RNA m^6^A represents a new layer of the regulatory mechanism controlling gene expression [19]. Accumulating data suggested that m^6^A modulators are dysregulated in various cancers [19, 20]. In this study, we found that m^6^A eraser ALKBH5 was highly expressed in MM, and increased ALKBH5 expression was correlated with a poor prognosis in MM patients. Using various HMCLs and xenograft models, we demonstrated that ALKBH5 and its demethylation activity were required for myeloma cell growth and survival. RNA-seq, m^6^A-seq, and ALKBH5-RIP assay identified TRAF1 as a direct target of ALKBH5. We showed that ALKBH5 positively regulated *TRAF1* mRNA stability in an m^6^A-dependent manner. Further functional studies validated that TRAF1 was a pivotal contributor to the oncogenic effect of ALKBH5 in MM. Of course, other potential targets of ALKBH5 may also contribute to the overall function of ALBKH5 in MM, which needs to be investigated systematically. Additionally, we examined the mechanisms underlying the MM-promoting effects of the ALKBH5-TRAF1 axis. Activation of NF-κB and MAPK signaling pathways plays a critical role in tumorigenesis in MM [59]. We demonstrated that as downstream targets of TRAF1, the key components of NF-κB and MAPK pathways could also be indirectly regulated by ALKBH5 in MM cells. Besides these mechanisms, other undiscovered tumor-promoting targets of TRAF1 might exist in MM cells, which requires systematic exploration.

The current study also provides insights into the expression profile of the other canonical m^6^A writers and erasers (METTL3, METTL14, WTAP, and FTO) and their impacts on MM proliferation. Several other reported m^6^A regulators were not covered in this study. However, we found YTHDF2 was a crucial reader protein that mediates the effect of ALKBH5 on TRAF1 transcripts. YTHDF2 was dysregulated in various human malignancies and played an oncogenic or tumor-suppressive role by facilitating the degradation of m^6^A-modified transcripts [60–66]. The role of YTHDF2 in MM has not yet been elucidated. Therefore, it needs to be investigated in the future. The current study mainly focused on the effects of ALKBH5 on myeloma cell growth and survival. RNA m^6^A modification was also found to be involved in the self-renewal of cancer stem cells, tumor metastasis, and drug response/resistance [20, 21], which encourages us to study other effects of ALKBH5 (or other regulators) on MM for a comprehensive understanding of the roles of m^6^A modification in MM.

Recent studies have linked ALKBH5 overexpression to epigenetic changes in some cancers [26, 67, 68]. For example, Wang et al. demonstrated that histone demethylase KDM4C reduced the H3K9me3 (trimethylation of lysine 9 on histone H3) level and increased the chromatin accessibility of ALKBH5 locus, that in turn promoted the transcription of ALKBH5 in AML cells [26]. Thus, we sought to investigate the upstream regulators of ALKBH5 in MM by studying the correlation between ALKBH5 expression and primary/secondary genetic events in MM. Although ALKBH5 was expressed at a high level in MM cluster with MMSET translocation, it might not be regulated by methyltransferase MMSET. Also, the expression of ALKBH5 was significantly higher in patients with IgH translocations compared with those with hyperdiploidy. However, tumor-promoting effects of ALKBH5 existed across our MM cell lines with various genetic characteristics (Fig. S9I), including RPMI-8226 that had no IgH translocation but displayed the highest ALKBH5 expression. Thus, the correlation between ALKBH5 expression and IgH translocation warrants an in-depth investigation. Furthermore, the dataset GSE39925 [30] showed that ALKBH5 expression was higher in the PCL group than the MM group, but we found that ALKBH5 deletion had a relatively moderate inhibitory effect on the cellular growth of JJN-3, a PCL cell line, compared with the MM cell lines, suggesting that ALKBH5 overexpression might not be the key driver for MM progression to PCL. Therefore, further investigation is needed into other potential upstream regulators of ALKBH5 in MM and the exact correlation between ALKBH5 expression and MM disease evolution.

In summary, the current study uncovered the importance of ALKBH5-mediated mRNA m^6^A demethylation in MM pathogenesis. ALKBH5 promoted MM cell survival and proliferation via posttranscriptional regulation of TRAF1 expression, which activated NF-κB and MAPK signaling pathways. Given the critical role of ALKBH5 in MM tumorigenesis, targeting ALKBH5 is a promising therapeutic strategy for treating MM patients.

## Methods

### Primary samples

All samples from newly diagnosed MM patients were collected from BM aspirations after obtaining informed consent. CD138 + MM cells were isolated using CD138 beads (Miltenyi Biotec, Germany). PBMCs from healthy donors were purified using Ficoll (Basal Media, Shanghai, China). All experiments using primary human samples were conducted in accordance with the ethical regulations and were approved by the ethics committees of the First Affiliated Hospital, School of Medicine, Zhejiang University, Hangzhou, China.

### Human MM cell line xenograft models

CAG, RPMI-8226, and U266 cells were transduced with indicated lentiviruses and selected using 2 μg/mL puromycin for 2 days. 5 × 10^5^ selected cells were injected via the tail vein into 4- to 5-week-old NCG mice (GemPharmatech, Nanjing, China). In vivo MM growth was monitored via chemiluminescence imaging of mice following intraperitoneal injection with luciferin (Promega, Madison, WA, USA) every 5 days. The mice were randomly assigned to the experimental groups. All animal experiments were approved by and conducted in accordance with the guidelines of the Animal Experimental Ethical Committee of the First Affiliated Hospital, School of Medicine, Zhejiang University.

### Quantification and statistical analysis

The quantification data were presented as mean ± standard deviation (SD) of three independent experiments. The log-rank (Mantel-Cox) test was performed for Kaplan–Meier survival analysis. All the other data were analyzed using the two-tailed Student’s *t*-test. Statistical analyses were performed using GraphPad Prism 9.0.

Additional methods are provided in supplemental Methods.

## Supplementary information

Supplemental materials
