# Supplementary material for: RNA demethylase ALKBH5 promotes tumorigenesis in multiple myeloma via TRAF1-mediated activation of NF-κB and MAPK signaling pathways
Source: Oncogene. 2021 Nov 10;41(3):400–13. doi: 10.1038/s41388-021-02095-8 (PMC8755544; doi:10.1038/s41388-021-02095-8)
Supplement: Supplementary file 1 — Supplemental materials [file 41388_2021_2095_MOESM1_ESM.pdf]

RNA demethylase ALKBH5 promotes tumorigenesis in multiple myeloma via TRAF1-mediated activation of NF-κB and MAPK signaling pathways

Supplemental Materials Include

(1) Supplemental Methods

(2) Supplemental Figures S1-S9 and Legends

(3) Supplemental Tables S1-S3

(4) Reference used in Supplemental Material

Supplemental Methods

Cell lines

The human myeloma cell lines (RPMI-8226, CAG, U266, MM1.S, NCI-H929, AR-P1, and JJN-3) were obtained as previously described [1] and maintained in RPMI-1640 supplemented with 10% FBS. 293T cells were purchased from the Cell Bank of the Chinese Academy of Science and cultured in DMEM supplemented with 10% FBS. All human cell lines have been authenticated using STR profiling and were tested for mycoplasma contamination on a regular basis. All cells were cultured in a humidified atmosphere of 5% CO_2 at 37°C.

Lentivirus production and infection

The pGLV-H1-GFP-Puro– or pGLV-U6-Luc-Puro–based lentiviral shRNAs against ALKBH5, TRAF1, METTL3, METTL14, WTAP, FTO, and YTHDF2 were designed and constructed by GenePharma Bio-Tech (Shanghai, China). The overexpression of ALKBH5 and TRAF1 were achieved using the lentiviral expression pLV-EF1aF-Puro vector by GenePharma Bio-Tech (Shanghai, China). Hairpin-resistant ALKBH5-WT and ALKBH5-H204A were generated by the site-directed synonymous mutagenesis at the shA5#2-targeted sequence to rescue shRNA-mediated KD, with the hairpin target sites (see Table S1) mutated into GACCGTGCTGAGCGGCTACGC. The inducible lentiviral shRNA (shA5_Tet-on and control) were obtained from Genechem Biotech (Shanghai, China). To establish stable cell lines, the positive infected cells were selected with 2 μg/mL puromycin (InvivoGen, San Diego, CA, USA) after 96 h of lentiviral infection. For inducible shALKBH5, 0.5–4 μg/mL doxycycline (MedChemExpress, Princeton, NJ, USA) was added to induce the expression of shRNA. The target sequences for shRNAs are listed in Supplementary Table S1.

Cell proliferation and colony formation assay

For myeloma cell proliferation assays, the cells were seeded into 96-well plates at the density of 2000 cells/100 μL per well in five replicates. The cell viability was assessed by Cell Count Kit-8 (CCK-8) every 24 or 48 h following the manufacturer's protocols (Dojindo, Japan). The colony formation assays were conducted as described earlier with some modifications [2, 3]. Briefly, 5000 transduced MM cells per well were seeded in triplicate into 6-well plates that contained the complete growth medium supplemented with 0.7% and 0.35% of melted soft agar in the lower and upper layers, respectively. A layer of the growth medium was maintained over the upper layer of agar to prevent desiccation. The stained colonies were counted using AlphaView software after approximately 3 weeks of incubation.

Flow cytometry analysis

For cell apoptosis analysis, the cells were stained with Annexin V and PI before flow cytometry analysis following the manufacturer's protocols (Dojindo, Japan). For the EdU (5-ethynyl-2′-deoxyuridine) cell proliferation assay, the cells were cultured with 50 μM EdU at 37°C for 2 h. The cells were then fixed, permeabilized, and stained following the manufacturer's protocols (Ribobio, China). For the cell cycle analysis, the cells were fixed and then stained with PI staining buffer (Multisciences Biotech, China) for 30 min at room temperature. For detecting CD138+ cells, APC-anti-human CD138 antibodies were used (Biolegend, CA, USA). All flow

cytometric analyses were performed on a BD FACSCanto II flow cytometer, and data were analyzed with FlowJo or ModFit software.

Enzyme-linked immunosorbent assay

A Human Kappa Enzyme-linked Immunosorbent Assay (ELISA) Kit (Bethyl Laboratories, Montgomery, TX, USA) was used for detecting human kappa light chain in serum following the manufacturer's protocols. Briefly, 100 μL of kappa standard and diluted mice serum sample were added to designated wells pre-adsorbed with an anti-human kappa antibody. After sample binding, unbound proteins and molecules were washed off. Then, detection antibody, horseradish peroxidase (HRP) solution, and 3,3',5,5'-tetramethylbenzidine (TMB) substrate were added to catalyze a colorimetric reaction. Finally, the absorbance at 450 nm was measured after adding the stop solution. The kappa concentrations in the test samples were quantified by interpolating their absorbance from the standard curve. Each sample was tested in duplicate.

m^{6}A dot blot assay

Total RNA was extracted from cells using TRIzol (TaKaRa, Japan) and quantified using a NanoDrop spectrophotometer. The m^{6}A dot blot assay was performed following a published protocol with some modifications [4]. Briefly, RNA samples were loaded onto the Amersham Hybond-N+ membrane (GE Healthcare, Madison, WI, USA) by vacuuming the Bio-Dot Apparatus and then UV cross-linked to the membrane. The membrane was blocked with 5% nonfat dry milk (dissolved in 1× TBS) for 1 h and incubated with an anti-m^{6}A antibody (Synaptic Systems, Germany) overnight at 4°C. Then membranes were rinsed with 1× TBST and incubated with HRP-conjugated goat anti-rabbit IgG for 1 h at room temperature. Chemiluminescent detection was performed with an ECL kit (Fdbio Science, China).

RNA-seq and data analysis

The RNA-seq was sequenced and analyzed by Novogene (Beijing, China). Briefly, mRNA was purified from total RNA using poly-T oligo-attached magnetic beads. An NEBNext Ultra RNA Library Prep Kit for Illumina (New England Biolabs, Beverly, MA, USA) was used for library preparation. RNA libraries were sequenced on an Illumina Novaseq 6000 platform with paired-end reads (150-bp read length). Paired-end clean reads were aligned to the human genome version GRCh38 using Hisat2 v2.0.5. The featureCounts v1.5.0-p3 was used to count the read numbers mapped to each gene. The FPKM of each gene was calculated based on the length of the gene and reads count mapped to this gene. Gene expression (RPKM) was calculated using RSEM. The average gene expressions of three biological replicates were used for the analysis. Differential expression analysis was performed using the DESeq2 R package (1.20.0). Genes with an adjusted P value <0.05 found by DESeq2 were considered as differentially expressed. We used the clusterProfiler R package to test the statistical enrichment of differentially expressed genes in Kyoto Encyclopedia of Genes and Genomes (KEGG) pathways [5].

m^{6}A-seq and data analysis

For m^{6}A-seq, poly (A) mRNA was purified from 100 μg total RNA using a Dynabeads mRNA kit (Thermo Fisher Scientific, Waltham, MA, USA). m^{6}A-IP and library preparation were performed following the reported protocol [6]. Briefly, fragmented mRNA (~100 nt) was incubated for 2 h at

4°C with an anti-m^{6}A polyclonal antibody (Synaptic Systems, Germany) in the immunoprecipitation experiment. Then, immunoprecipitated mRNAs or input was used for library construction with an NEBNext Ultra RNA Library Prep Kit for Illumina (New England Biolabs, Beverly, MA, USA). The library preparations were sequenced on an Illumina Novaseq 6000 platform with a paired-end read length of 150 bp following the standard protocols. After mapping reads to the GRCh38 human reference genome, the exomePeak R package (version 2.16.0) was used for the m^{6}A peak identification in each anti-m^{6}A immunoprecipitation group with the corresponding input samples serving as a control. The q-value threshold of an enrichment of 0.05 was used. The m^{6}A-enriched motifs in each group were identified using HOMER (version 4.9.1). Differential peak calling was performed using the exomePeak R package with parameters of P value less than 0.05 and a fold change more than 1. The m^{6}A-seq and following data analyses were carried out by Novogene (Beijing, China).

The RNA-seq data and m6A-seq data have been deposited into the Gene Expression Omnibus database (accession number GSE180215)

Dual-luciferase reporter assay

The wild-type and mutant TRAF1 3′-UTR sequences (NM_005658.5, 1725–2455) were amplified and cloned in the pmirGlo vector to construct a dual-luciferase reporter (TRAF1-3′UTR-WT and TRAF1-3′UTR-Mut) by GenePharma (Shanghai, China). For mutant reporter plasmids, adenosine (A) in m^{6}A motifs (at positions 1777, 2019, and 2429) were replaced by cytosine (C). 293T cells transduced with indicated lentivirus were seeded in 24-well plates and transfected with a dual-luciferase vector fused with the wild-type or mutated TRAF1-3′-UTR. The activities of firefly luciferase and Renilla luciferase in each well were detected using a dual-luciferase reporter assay system (Promega, Madison, WA, USA) 48 h after treatment.

Quantitative RT-PCR

Total RNA was isolated from MM cells using TRIzol (TaKaRa, Japan) following the manufacturer's protocol. For detecting mRNA expression, 1000 ng of total RNA was reverse-transcribed into cDNA in a total reaction volume of 20 μL with the HiScript II Q RT SuperMix for qPCR (plus gDNA wiper) Kit (Vazyme, China). Quantitative real-time PCR was then conducted with 2 μL of diluted cDNA (with fourfold dilution) using SYBR Green qPCR master mix (Vazyme, China) on a Bio-Rad real-time PCR machine following the manufacturer's protocols. GAPDH was used as endogenous control. Each sample was run in triplicate. The $2^{-\Delta\Delta Ct}$ method was used to normalize the expression to GAPDH. The primers used for qPCR analysis are listed in Supplementary Table S2.

m^{6}A-IP-qRCR

For m^{6}A immunoprecipitation (IP), a Magna MeRIP m^{6}A Kit (Millipore, Billerica, MA, USA) was used following the manufacturer's protocols. Briefly, fragmented total RNA was incubated with m^{6}A antibody- or mouse IgG-conjugated Protein A/G Magnetic Beads for 2 h at 4°C; one tenth of fragmented RNA was saved as input control. After washing four times with IP buffer, the beads were eluted twice with 100 μL of elution buffer. Then, the eluted RNA was purified using an RNeasy mini kit (Qiagen, Germany). MeRIPed RNAs and input RNAs were further analyzed by

qPCR using primers listed in Table S2. The comparative Ct (ΔΔCt) method was used to compare m^6A enrichment of each sample. First, Ct values of sample were normalized with anti-m^6A and the samples with negative control IgG to input: $\Delta Ct = Ct_{IP} - (Ct_{input} - \text{Log2 [Input Dilution Factor]})$ (input dilution factor was 10 for using 10% input sample). Then, the ΔCt values of the sample with anti-m^6A were normalized to the sample with negative control IgG: $\Delta\Delta Ct = \Delta Ct_{m6A} - \Delta Ct_{IgG}$). The fold enrichment of the sample with the anti-m^6A antibody over the negative control mouse IgG control was calculated: fold enrichment = $2^{-\Delta\Delta Ct}$.

RIP-qPCR

For ALKBH5 RIP, RNA immunoprecipitation was performed with a Magna RIP RNA-Binding Protein Immunoprecipitation Kit (Millipore, Billerica, MA, USA) following the manufacturer's protocols. Briefly, magnetic beads coated with 5 μg of antibodies against ALKBH5 (Sigma–Aldrich, Germany) and rabbit IgG were incubated with cell lysates overnight at 4°C. The immunoprecipitated complexes were washed six times and then subjected to proteinase K digestion and RNA purification. The relative enrichment of immunoprecipitated RNA was analyzed by qPCR and normalized to input. After normalizing Ct values of the sample immunoprecipitated with anti-ALKBH5 and the samples with negative control (IgG) to input (ΔCt): $\Delta Ct = Ct_{IP} - (Ct_{input} - \text{Log2 [Input Dilution Factor]})$ (input dilution factor was 10 for using 10% input sample), the percentage of input for each IP sample was calculated: %Input = $2^{(-\Delta Ct \text{ [normalized IP]})}$.

RNA decay assay

MM cells were treated with mRNA transcription inhibitor actinomycin D (MCE, HY-17559) at a final concentration of 5 μg/mL and collected at indicated time points. Total RNA was extracted using TRIzol and analyzed using qRT-PCR. GAPDH was used as endogenous control. The half-life of mRNA was estimated as previously described [7].

Western blotting

The cells were lysed in RIPA mixed with protease and phosphatase inhibitor cocktail (Thermo Fisher Scientific, Rockford, IL, USA) for 15 min on ice. The cell extracts were centrifuged at 12,000*g* for 15 min, and the supernatants were then collected. The protein was quantified using a Bicinchoninic Acid (BCA) Protein Assay Kit (KeyGEN, China). Proteins were separated in SDS-PAGE gel and transferred onto 0.45-μm PVDF membranes (Millipore, Billerica, MA, USA) which were blocked with 5% nonfat milk dissolved in 1× TBST for 1h, incubated sequentially with primary and secondary antibodies, and detected by immunoblotting with an ECL kit (Fdbio Science, China). The anntibodies used for Western blotting are listed in Supplementary Table S3. GAPDH and/or Cyclophilin B were used as loading controls.

Public database analysis

The publicly available datasets of gene expression used in the study included Amazonia! atlas (amazonia.transcriptome.eu), the Cancer Cell Line Encyclopedia (CCLE) data set, Oncomine database, GenomicScape (www.genomicscape.com) atlases or gene sets, and datasets under the NCBI Gene Expression Omnibus (GEO) accession numbers GSE5900, GSE39925, GSE4581, GSE9782, GSE26760, GSE19784, GSE57317, GSE82307, GSE31161, GSE15695, GSE2658,

GSE29147, GSE29148, GSE57863, and GSE24746. They were used for survival analysis, gene expression analysis, and/or Gene Set Enrichment Analysis (GSEA) [8].

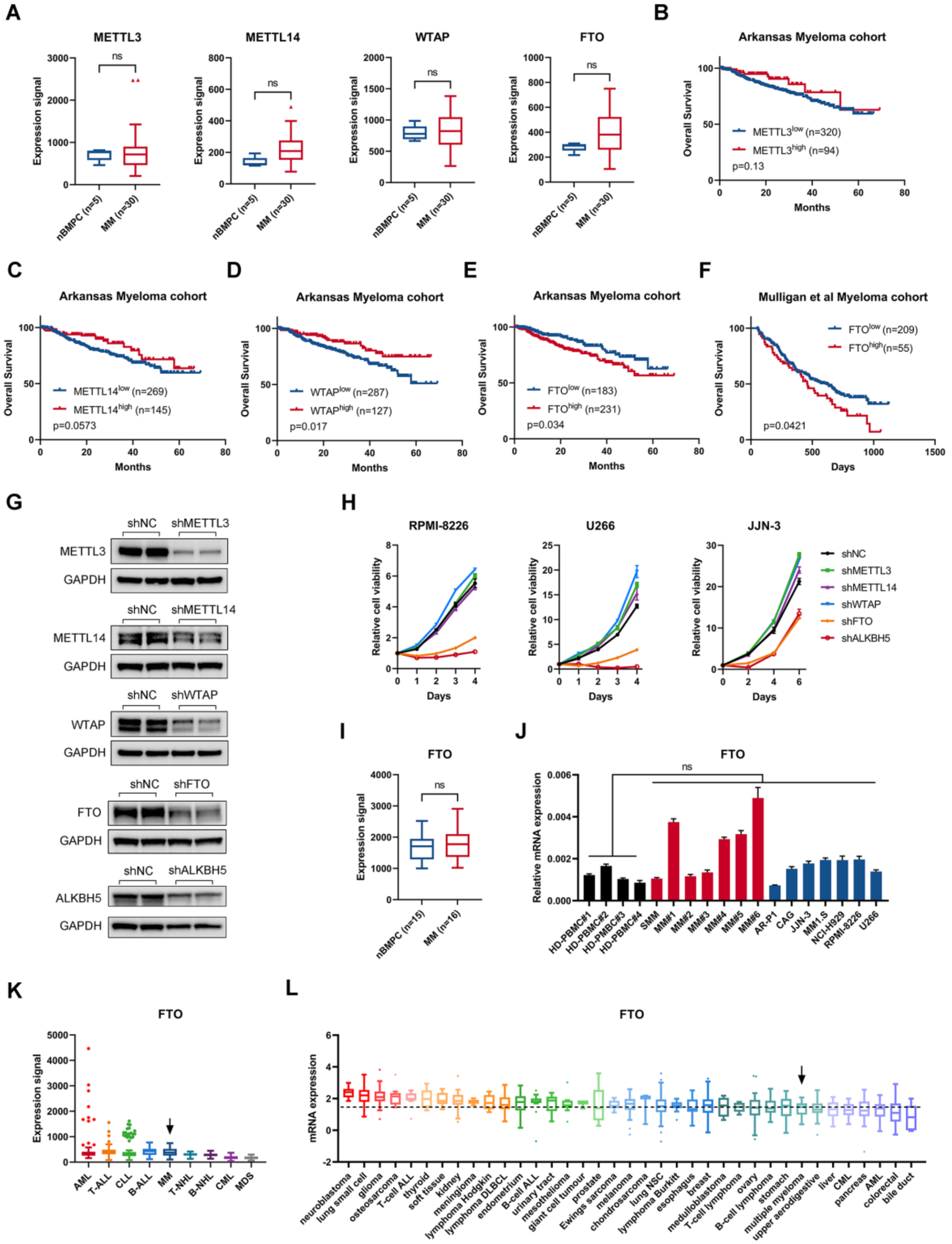

Figure S1. Expression of m^{6}A modifiers in MM and their impacts on the prognosis of MM patients.

(A) Normalized expression signal of METTL3, METTL14, WTAP, and FTO in normal bone marrow plasma cells (nBMPCs) and MM cells according to Amazonia! Atlas (MM and MicroE, U133P). **(B–E)** Correlation between the survival of MM patients and METTL3 (B), METTL14 (C), WTAP (D), and FTO (E) expression in the Arkansas myeloma dataset (GSE4581) [9]. Overall patient survival in groups of high and low expression was analyzed by Kaplan–Meier survival

curve. Cutoff values were determined by the maximum standardized log-rank statistics. The *P*-value was detected using the log-rank test. **(F)** Kaplan–Meier plots of overall survival in Mulligan et al. [10] myeloma cohorts for patients with MM (GSE9782), stratified on the basis of FTO expression (high and low). The optimal cutoff value was determined by the maximum standardized log-rank statistic. The *P*-value was detected using the log-rank test. **(G)** Western blots showing the knockdown (KD) efficiency of indicated proteins in MM cells by shRNAs. **(H)** Growth curves of RPMI-8226, U266, and JJN-3 cells transduced with indicated lentiviruses. **(I)** Normalized expression signal of FTO in normal bone marrow plasma cells (nBMPCs) and MM cells according to Amazonia! Atlas (MM - Tarte et al. [11] HuGeneFL) **(J)** qRT-PCR analysis of FTO expression in normal peripheral blood mononuclear cells (PBMCs) from healthy donors, primary cells derived from patients with MM, and human myeloma cell lines, using GAPDH as the reference. **(K)** Normalized expression signal of FTO in various hematological malignancies, according to Amazonia! Atlas. AML, Acute myeloid leukemia; T-ALL, T-cell acute lymphoblastic leukemia; CLL, chronic lymphocytic leukemia; B-ALL, B-cell acute lymphoblastic leukemia; T-NHL, T-cell non-Hodgkin lymphoma; B-NHL, B-cell non-Hodgkin lymphoma; CML, chronic myeloid leukemia; MDS, myelodysplastic syndromes. **(L)** mRNA expression of FTO in MM and other indicated cancer cell lines, according to the Cancer Cell Line Encyclopedia data set. T-cell ALL, T-cell acute lymphoblastic leukemia; DLBCL, diffuse large B-cell lymphoma; B-cell ALL, B-cell acute lymphoblastic leukemia; lung NSC, non-small cell lung cancer; CML, chronic myeloid leukemia; AML, acute myeloid leukemia. $^{*}P < 0.05$, $^{**}P < 0.01$, $^{***}P < 0.001$, and $^{****}P < 0.0001$ (*t*-test). ns, No significance. Error bars denote mean ± SD.

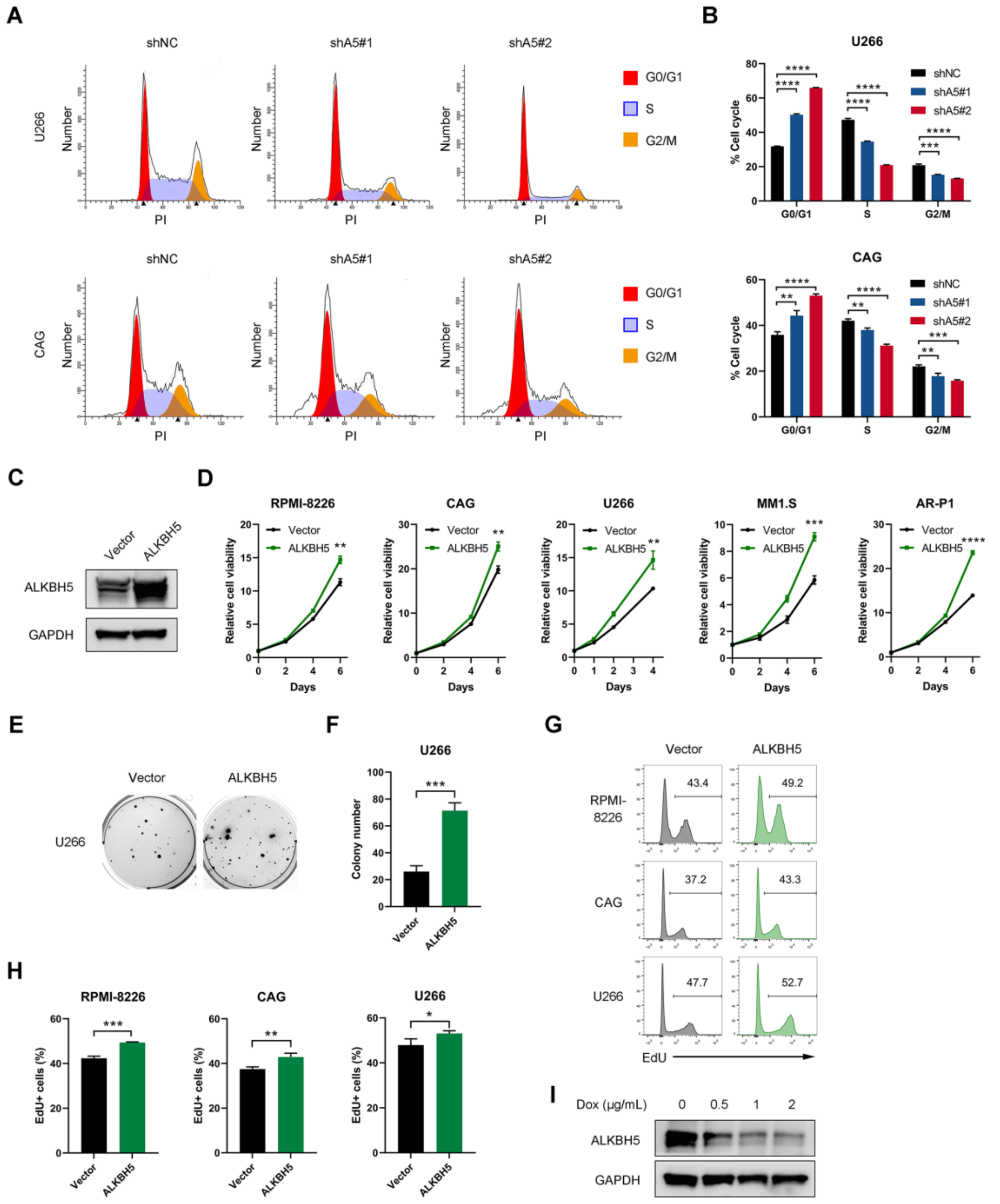

Figure S2. ALKBH5 played a pro-proliferative and -survival role in MM cells *in vitro*.
(A, B) Cell cycle analysis of MM cells with or without ALKBH5 KD. Representative flow cytometry plots (A) and statistics of the percentage of cells in indicated cell cycle phase (B). **(C)** Immunoblot of ALKBH5 in U266 cells with or without ALKBH5 overexpression. **(D)** Proliferation of MM cells transduced with indicated lentiviruses as determined by CCK-8. **(E, F)** Representative colony images (E) and statistics of colony counts (F) of U266 cells with or without ALKBH5 overexpression. **(G, H)** EdU assay of MM cells with or without ALKBH5 overexpression. Representative flow cytometry plots (G) and statistics of the percentage of EdU-positive cells in MM cells (H). **(I)** Western blot of ALKBH5 after inducible ALKBH5 KD by doxycycline (Dox) at indicated concentrations. $^{*}P < 0.05$, $^{**}P < 0.01$, $^{***}P < 0.001$, and $^{****}P < 0.0001$ (*t*-test). ns, No significance. Error bars denote mean ± SD.

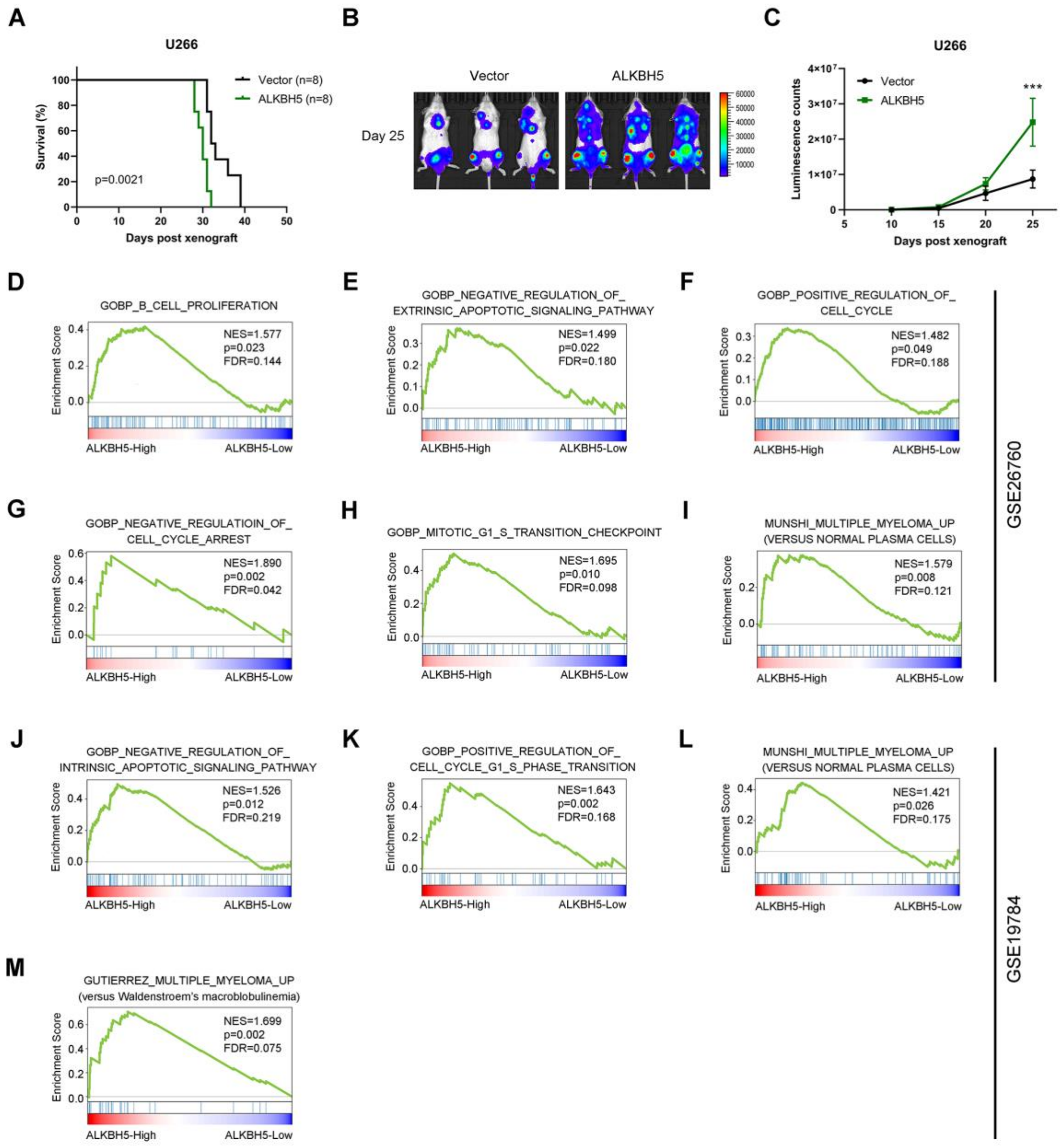

Figure S3. ALKBH5 promoted MM cell growth *in vivo*.

(A–C) Kaplan–Meier curve for the survival (A), representative image (25 days post-xenograft; B), and summary of chemiluminescence signals (C) of mice implanted with luciferase-labeled U266 cells with or without ALKBH5 overexpression (n = 8/group). **(D–M)** GSEA plots showing enrichment of indicated gene sets in ALKBH5-High (top 50%) versus ALKBH5-Low (bottom 50%) groups from MM patient cohorts GSE26760 [12] and GSE19784 [13] (J-M). $^{*}P < 0.05$, $^{**}P < 0.01$, $^{***}P < 0.001$, and $^{****}P < 0.0001$ (t-test). ns, No significance. Error bars denote mean ± SD.

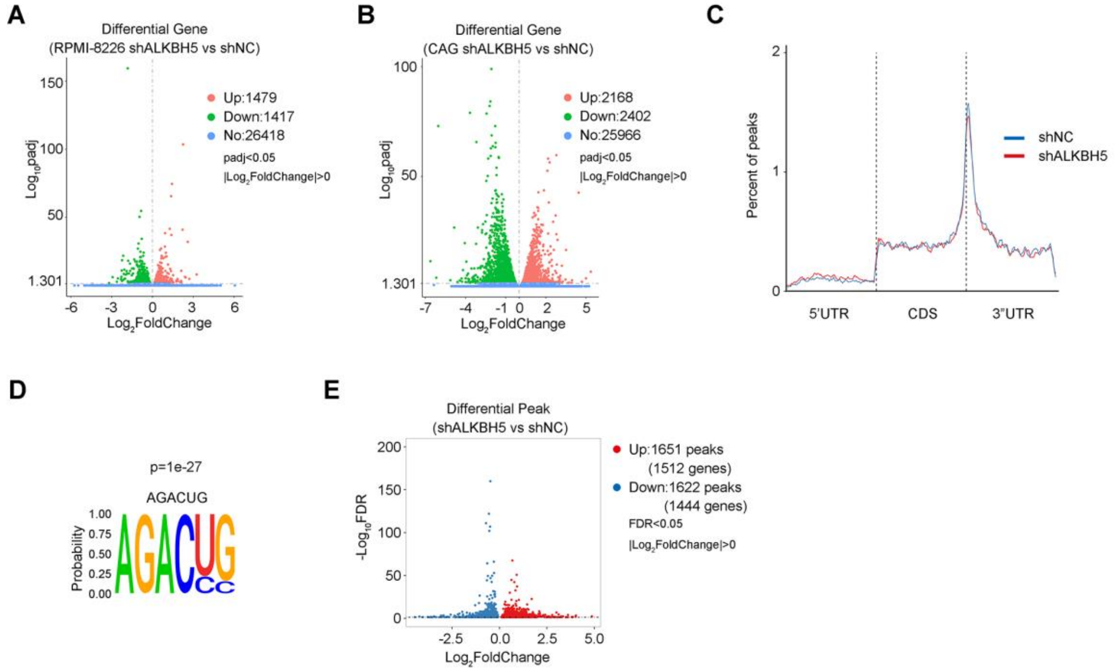

Figure S4. Analysis of RNA sequencing and m^{6}A sequencing of MM cells with or without ALKBH5 knockdown.

(A, B) Volcano plot showing differential expression of genes upon ALKBH5 KD in RPMI-8226 (A) and CAG (B) by RNA-seq. **(C)** m^{6}A peak distribution along a normalized transcript. 5'UTR, 5'-untranslated region; CDS, coding sequence; 3'UTR, 3'-untranslated region. **(D)** Sequence motifs enriched in m^{6}A peaks identified by HOMER. **(E)** Volcano plot of differential m^{6}A peaks in MM cells with or without ALKBH5 KD.

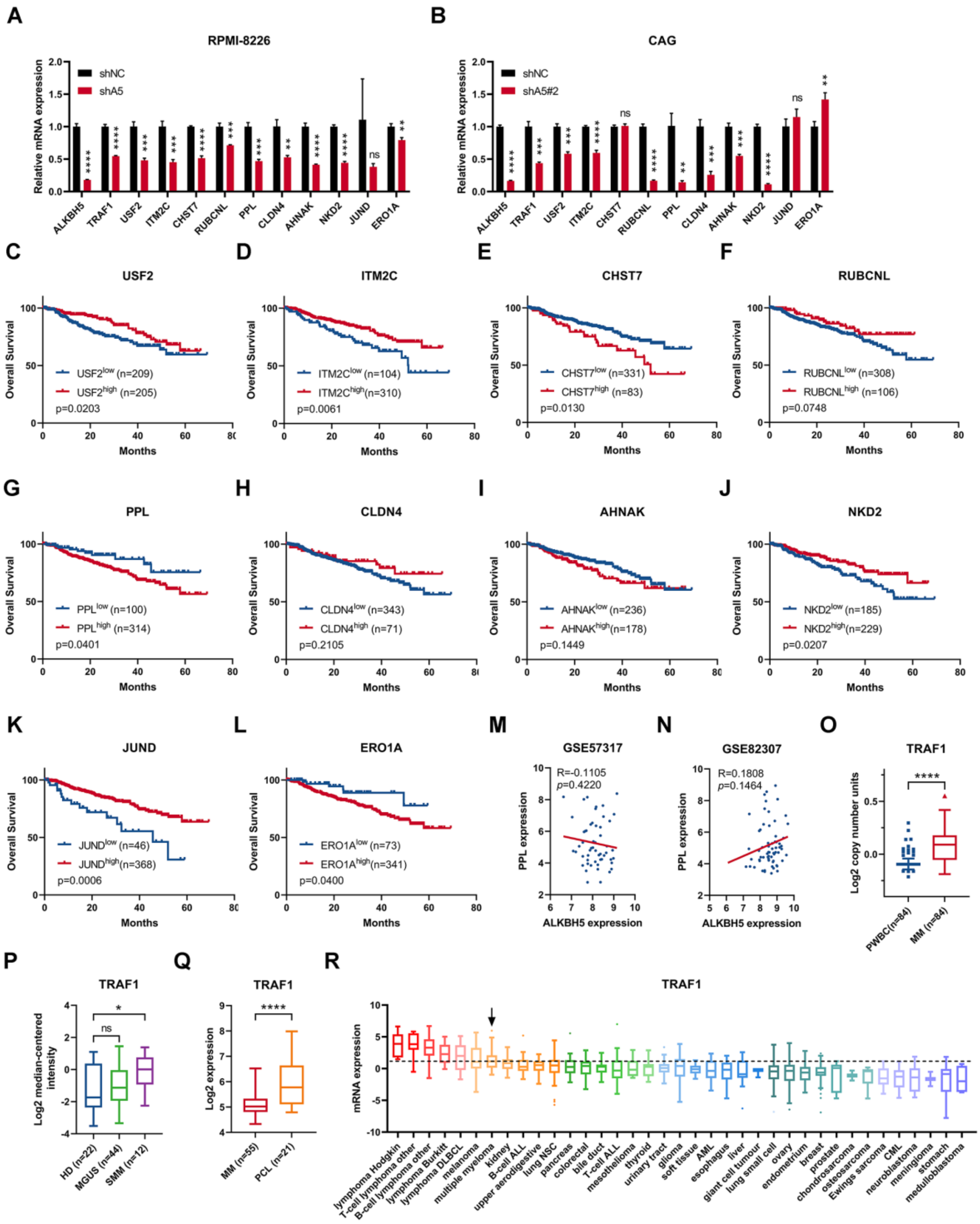

Figure S5. Identification of potential downstream targets of ALKBH5 in MM cells.
(A, B) qPCR analysis showing expression change of potential targets of ALKBH5 upon ALBH5 KD in RPMI-8226 (A) and CAG (B) cells. **(C–L)** Kaplan–Meier survival analysis of potential targets of ALKBH5 in the Arkansas myeloma dataset (GSE4581) [9]. Optimal cutoff values were determined by the maximum standardized log-rank statistic. The *P*-value was detected using the log-rank test. **(M, N)** Pearson correlation between ALKBH5 and PPL mRNA expression in indicated datasets [14, 15]. **(O)** Copy number of TRAF1 in 84 paired normal and CD138+ purified multiple myeloma samples in Dickens et al. [16] Myeloma data set (GSE15695) according to

Oncomine database. **(P)** TRAF1 expression in samples from healthy donors (HD) or patients diagnosed with monoclonal gammopathy of undetermined significance (MGUS), smoldering MM (SMM) in Zhan et al. [17] myeloma dataset according to Oncomine database. **(Q)** TRAF1 expression in samples from patients diagnosed with MM and PCL in the dataset GSE39925 [18]. **(R)** TRAF1 mRNA expression in MM and other indicated cancer cell lines, according to the Cancer Cell Line Encyclopedia dataset. DLBCL, diffuse large B-cell lymphoma; B-cell ALL, B-cell acute lymphoblastic leukemia; lung NSC, non-small cell lung cancer; T-cell ALL, T-cell acute lymphoblastic leukemia. AML, Acute myeloid leukemia; CML, chronic myeloid leukemia. $^{*}P < 0.05$, $^{**}P < 0.01$, $^{***}P < 0.001$, and $^{****}P < 0.0001$ (*t*-test). ns, No significance. Error bars denote mean ± SD.

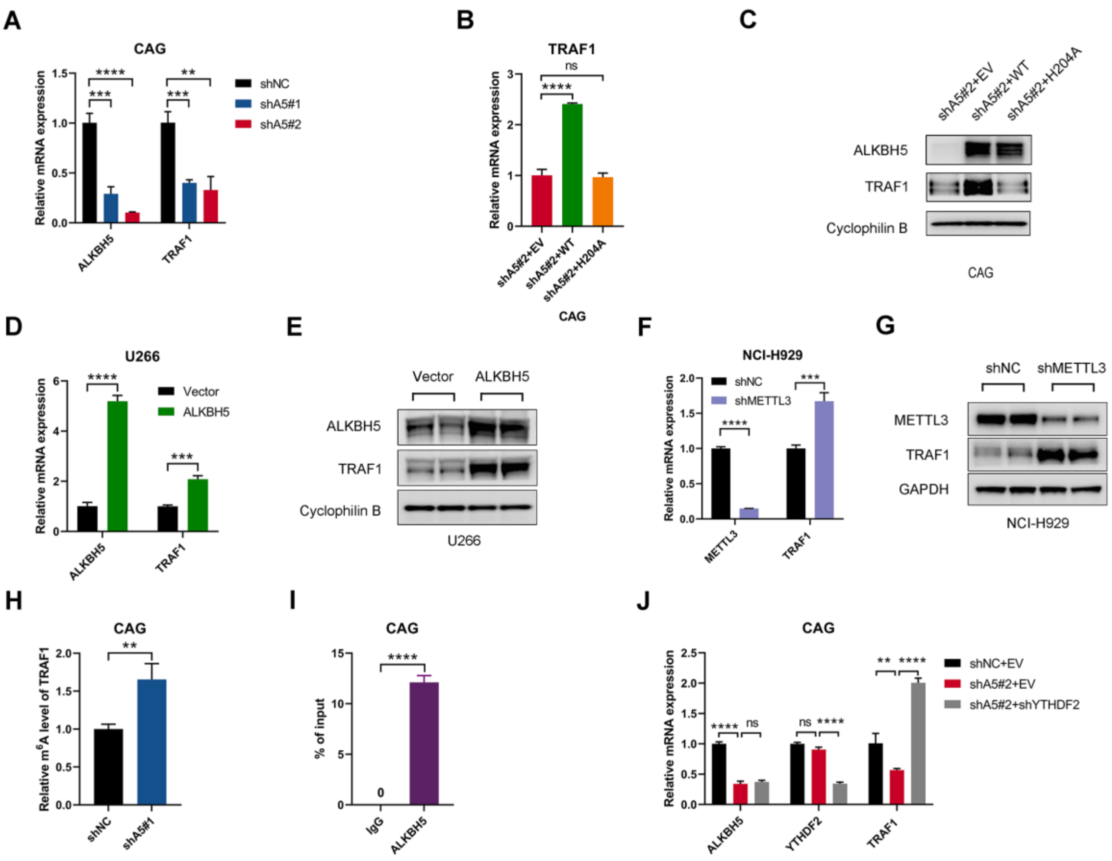

Figure S6. ALKBH5 regulated TRAF1 expression by altering its mRNA stability in an m6A-dependent manner.

(A) qRT-PCR analysis of ALKBH5 and TRAF1 mRNA levels in CAG cells transduced with indicated lentivirus. **(B)** Relative mRNA levels of ALKBH5 and TRAF1 CAG cells transduced with indicated lentiviruses. EV represents empty vector; WT represents wild-type ALKBH5; and H204A represents m^{6}A demethylase-inactive mutant. **(C)** Immunoblot for ALKBH5 and TRAF1 in CAG cells after transduction with indicated lentivirus. Cyclophilin B was used as a loading control. **(D, E)** Relative mRNA levels (F) and Western blots (G) of ALKBH5 and TRAF1 in U266 cells with or without ALKBH5 overexpression. **(F, G)** Relative mRNA levels (F) and Western blots (G) of METTL3 and TRAF1 in MM NCI-H929 cells with or without METTL3 KD. **(H)** m^{6}A-IP-qPCR analysis of m^{6}A enrichment on TRAF1 mRNA in CAG cells with or without ALKBH5 KD. **(I)** ALKBH5-RIP-qPCR validation of ALKBH5 binding to TRAF1 mRNA in CAG cells. **(J)** qRT-PCR analysis of ALKBH5, YTHDF2 and TRAF1 mRNA expression in MM cells transduced with indicated lentiviruses. EV represents empty vector. $^{*}P < 0.05$, $^{**}P < 0.01$, $^{***}P < 0.001$, and $^{****}P < 0.0001$ (t-test). ns, No significance. Error bars denote mean ± SD.

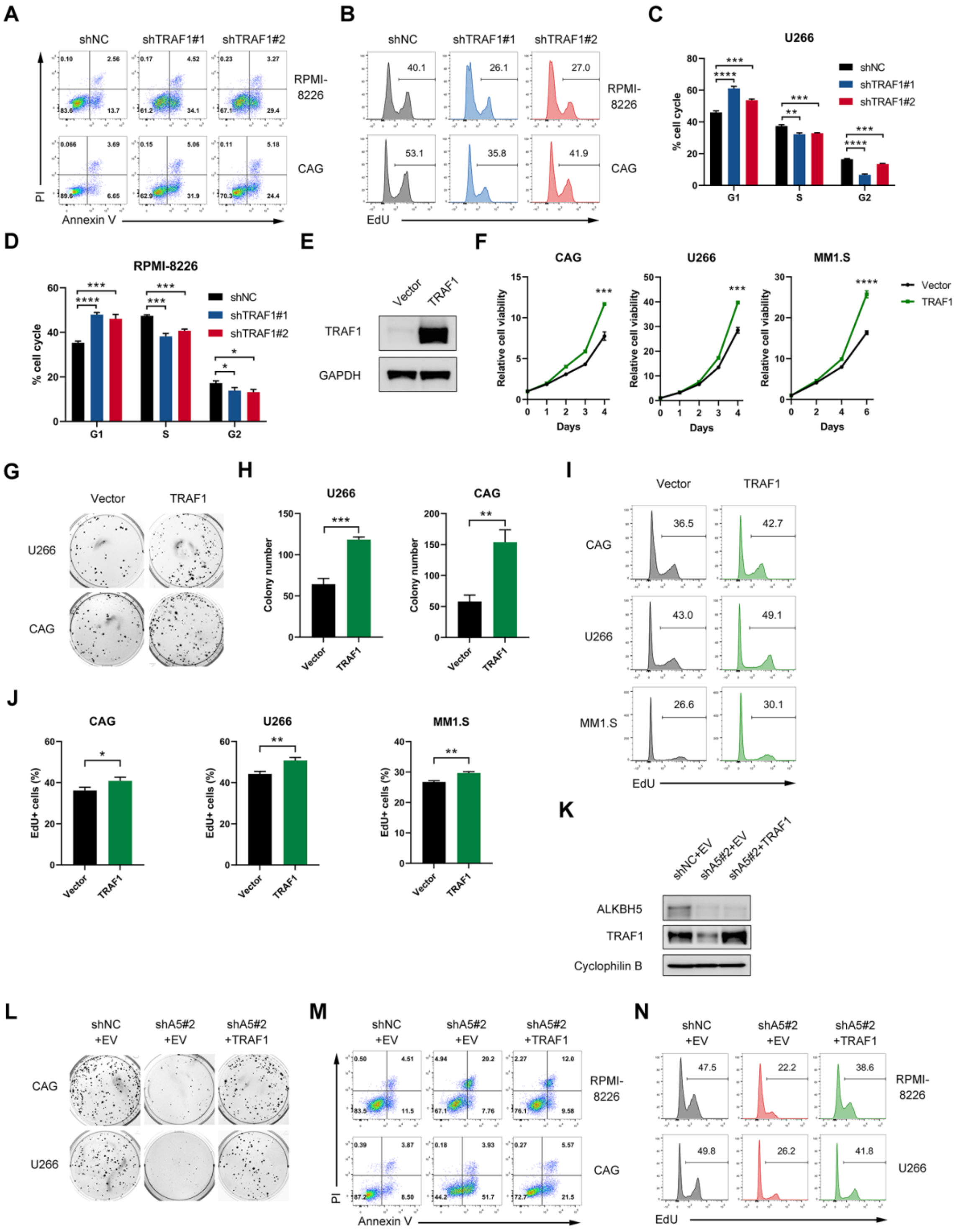

Figure S7. TRAF1 was a functionally important target of ALKBH5 in MM.
(A) Representative flow cytometry plots of the apoptosis analysis of MM cells upon TRAF1 KD (supplement to Figure 7E). **(B)** Representative flow cytometry plots of the percentage of EdU-positive cells in RPMI-8226 and CAG cells upon TRAF1 KD (supplement to Figure 7F). **(C, D)** Cell cycle analysis of U266 (C) and RPMI-8226 (D) cells with or without TRAF1 KD. **(E)** Immunoblot of TRAF1 in MM cells with or without TRAF1 overexpression. **(F)** Proliferation of MM cells CAG, U266, and MM1.S transduced with indicated lentiviruses as determined by

CCK-8. **(G, H)** Representative colony images (G) and statistics of colony counts (H) of MM cells with or without TRAF1 overexpression. **(I, J)** EdU (5-ethynyl-2′-deoxyuridine) assay of MM cells with or without TRAF1 overexpression. Representative flow cytometry plots (I) and statistics of the percentage of EdU-positive cells (J) in RPMI-8226, CAG, and U266. **(K–N)** Effects of TRAF1 restoration (K) on colony-forming capacity (L; supplement to Figure 7K), apoptosis (M; supplement to Figure 7L), and DNA synthesis (revealed by EdU; N; supplement to Figure 7M) of ALKBH5 KD MM cells. $^{*}P < 0.05$, $^{**}P < 0.01$, $^{***}P < 0.001$, and $^{****}P < 0.0001$ (*t*-test). ns, No significance. Error bars denote mean ± SD.

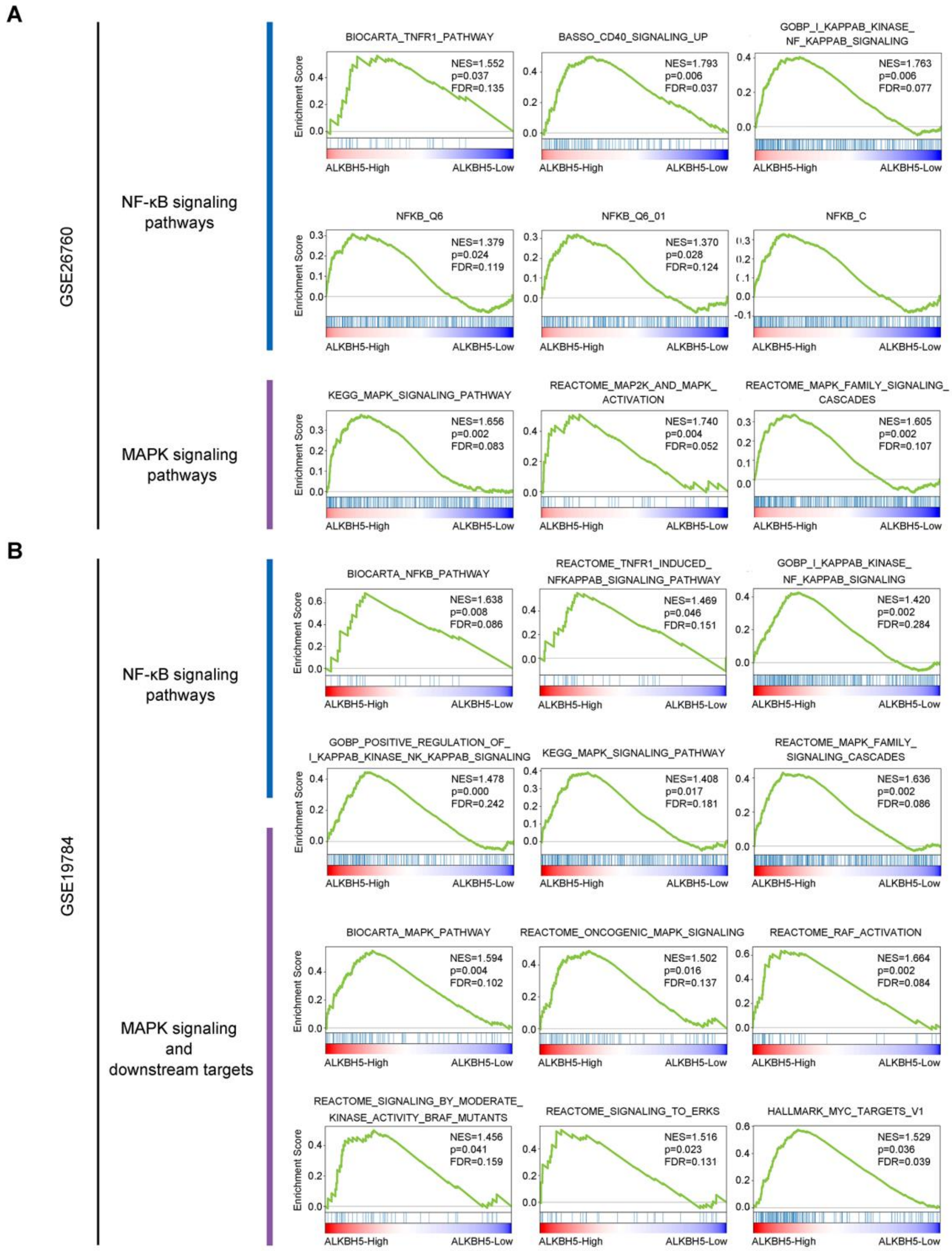

Figure S8. ALKBH5-TRAF1 regulated NF-κB and MAPK pathways in MM.

(A, B) GSEA plots showing the enrichment of gene sets of NF-κB signaling pathways, MAPK signaling pathways and MAPK downstream targets in ALKBH5-High (top 50%) versus ALKBH5-Low (bottom 50%) groups from MM patient cohorts GSE26760 [12] (A; supplement to Figure 8B) and GSE19784 [13] (B).

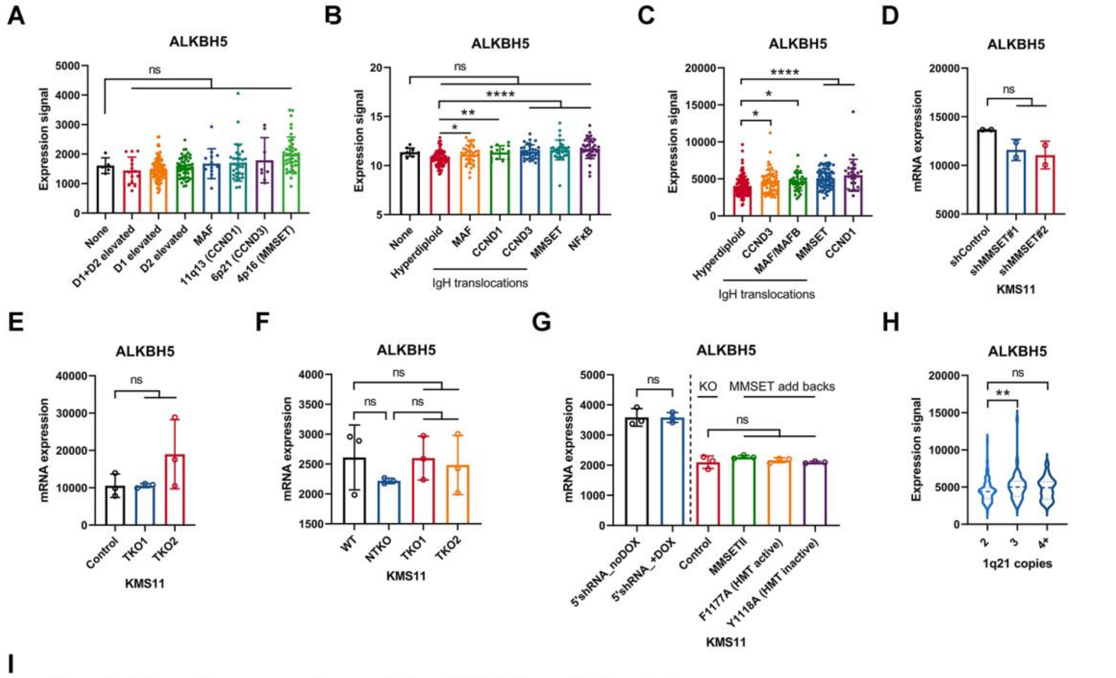

I

Characteristics of human myeloma cell lines (HMCLs) used in the study

Name	Disease	Translocations	Cyclin D type	1q21 copies	1p32 copies	Other genetic characteristics
RPMI-8226	MM	t(16;22) + t(8;22)	D2	4	4	$KRAS^{mut}$ + $TP53^{mut}$ +$TRAF3^{mut}$
CAG	MM	t(14;16) / MAF	D2	6	2	$TP53^{negative}$
U266	MM	t(11;14) / CCND1	D1+D2	3	2	$BRAF^{mut}$ + $RB1^{mut}$ + $TP53^{mut}$ + $TRAF3^{mut}$
MM1.S	MM	t(14;16) / MAF + t(8;14) / MYC	D2	NA	NA	$KRAS^{mut}$ + $TRAF3^{mut}$
NCI-H929	MM	t(4;14) / MMSET	D2	4	2	$NRAS^{mut}$
AR-P1	MM	t(14;16) / MAF	D2	8	4	$TP53^{negative}$
JJN-3	PCL	t(14;16) / MAF+ t(8;14) / MYC	D2	4	3	$TP53^{negative}$

Abbreviations: MM, multiple myeloma; PCL, plasma cell leukemia; NA, not available; mut, mutated.

Figure S9. ALKBH5 expression in MM subgroups with different genetic features.

(A) ALKBH5 expression in different genetic subgroups in Mulligan et al. [10] MM dataset (GSE9782). **(B)** Comparison of the expression levels of ALKBH5 in patients with MM bearing different genetic alterations based on the HOVON65/GMMG-HD4 cohort (GSE19784) [13]. **(C)** ALKBH5 expression levels in different genetic subgroups in Zhan et al. [9] cohort (GSE2658). **(D)** ALKBH5 expression by array of KMS11 cells stably transduced with a control vector in comparison to two independent shRNAs against MMSET (NSD2) according to GSE29147 [19]. **(E)** ALKBH5 expression of KMS11 cells with or without t(4;14) translocation knockout (TKO) according to GSE29148 [19]. **(F)** ALKBH5 expression of parental KMS11 cell lines (WT), KMS11 cells with the wild-type MMSET allele inactivated (NTKO), and two different clones with rearranged IgH-MMSET allele inactivated (TKO1 and TKO2) according to GSE57863 [20]. **(G)** ALKBH5 expression in KMS11 cells with (+Dox) or without (no Dox) MMSET knockdown (left panel); and in KMS11 cells with MMSET translocation knockout (control) or MMSET restoration by wild-type (MMSETII) or histone methyl-transferase (HMT) activity mutants (F1177A HMT active and F1118A HMT inactive) according to GSE24746 [21]. **(H)** Violin plots showing ALKBH5 expression levels in patients with MM having different 1q21 copies in the Zhan et al. [9] MM dataset (GSE2658). **(I)** Characteristics of human MM cells used in the study. Data sources: Keats Lab database (www.keatslab.org) and published articles [22-29]. $^{*}P < 0.05$, $^{**}P < 0.01$, $^{***}P < 0.001$, and $^{****}P < 0.0001$ (*t*-test). ns, No significance. Error bars denote mean ± SD. None, unclassified; 4p16 (MMSET), t(4;14)(p16;q32) or MMSET translocation; 6p21 (CCND3),

t(6;14)(p21;q32), or CCND3 translocation; 11q13 (CCND1), t(11;14)(q13;q32) or CCND1 translocation, MAF, t(14;16)(q32;q23)/t(14;20)(q32;q11), or MAF/MAFB translocations; D1, CCND1; D2, CCND2.

Table S1 (Related to Methods) List of target sequences for shRNAs

Name	Target Sequence (5' - 3')	Note
shNC	TTCTCCGAACGTGTCACGT	
shMETTL3	GCTGCACTTCAGACGAATTAT	
shMETTL14	GCATTGGTGCCGTGTTAAATA	
shWTAP	GCGAAGTGTCGAATGCTTATC	
shFTO	GGATGGGTTCATCCTACAACG	
shALKBH5#1	GACGTCCCGGGACAACTATAA	
shALKBH5#2	GACTGTGCTCAGTGGATATGC	also for shA5_Tet-on
shTRAF1#1	GGAACAAGGTCACCTTCATGC	
shTRAF1#2	GCACTTTCCTGTGGAAGATCA	
shYTHDF2	GGACGTTCCCAATAGCCAACT	

Table S2 (Related to Methods) List of primer sequences

Name		Sequence (5' - 3')	Note
GAPDH	Forward	TTGGTATCGTGGAAGGACTCA	
	Reverse	TGTCATCATATTTGGCAGGTTT	
ALKBH5	Forward	CGGCGAAGGCTACACTTACG	
	Reverse	CCACCAGCTTTTGGATCACCA	
TRAF1	Forward	TCCTGTGGAAGATCACCAATGT	
	Reverse	GCAGGCACAACTTGTAGCC	
TRAF1	Forward	AACTCAGAAGGGAGCTAGCCA	m^6A-IP qPCR
	Reverse	CCCTCAGTCTTGCTTGGATCA	
FTO	Forward	CGCATGGCAGCAAGCTAA	
	Reverse	GCAAACCATAGGCTGGGTCAT	
METTL3	Forward	CGGGTAGATGAAATTATTTGGG	
	Reverse	GATTTCCTTTGACACCAACC	
YTHDF2	Forward	CCAAGAGGAAGAAGAAAGTG	
	Reverse	AGTCCTAATTCTCTTGAAGGTC	
USF2	Forward	CTGTGATCCAAAATCCCTTCAGC	
	Reverse	GGTCTGTGGTCTGTACGGAC	
ITM2C	Forward	CGCAGACGTACATCATCCAGG	
	Reverse	CGGTAGGTGTCTTTCCCGTTG	
CHST7	Forward	ACACGGCCAATCTTACCACG	
	Reverse	CGGGTACTTTCGGCACTCG	
RUBCNL	Forward	CTCAACAGAGGTACACATGGTC	
	Reverse	TGTCAGTCTCAGGATATGGGG	
PPL	Forward	GCTGAAGACCGAGAATCCCG	
	Reverse	CCGCAGTAGCTCGTTGGTG	
CLDN4	Forward	GGGGCAAGTGTACCAACTG	
	Reverse	GACACCGGCACTATCACCA	
AHNAK	Forward	TACCCTTCCTAAGGCTGACATT	
	Reverse	TTGGACCCTTGAGTTTTGCAT	
NKD2	Forward	CCAGTGCGATGTCTCGGTG	
	Reverse	CCTTCCCGCAGTTGTCAAAGT	
JUND	Forward	TCATCATCCAGTCCAACGGG	
	Reverse	TTCTGCTTGTGTAAATCCTCCAG	
ERO1A	Forward	GACTGTGCTGTCAAACCATGT	
	Reverse	TCCAAGTCGTTCAGCTTGTTC	

Table S3 (Related to Methods) List of antibodies used in the study

Antibody	Source	Identifier
anti-GAPDH	Cell Signaling Technology	Cat#5174
anti-Cyclophilin B	Abcam	Cat#ab178397
anti-ALKBH5 (WB)	Abcam	Cat#ab195377
anti-ALKBH5 (IP)	Sigma-Aldrich	Cat#ABE547
anti-TRAF1	Cell Signaling Technology	Cat#4715
anti-m^6A (Dot Blot)	SYSY	Cat#202003
anti-m^6A (IP)	Merck Millipore	Cat#MABE1006
anti-METTL3	Abcam	Cat#ab195352
anti-METTL14	Abcam	Cat#ab220030
anti-WTAP	Abcam	Cat#ab195380
anti-FTO	Abcam	Cat#ab126605
anti-YTHDF2	Abcam	Cat#ab246514
anti-BRAF	Abcam	Cat#ab33899
anti-p-BRAF (Ser445)	Cell Signaling Technology	Cat#2696
anti-p-MEK1/2 (Ser221)	Cell Signaling Technology	Cat#2338
anti-p-p44/42 MAPK (Erk1/2) (Thr202/Tyr204)	Cell Signaling Technology	Cat#4370
anti-p-c-Fos (Ser32)	Cell Signaling Technology	Cat#5348
anti-c-Myc	Cell Signaling Technology	Cat#18583
anti-NF-κB1 p105/p50	Cell Signaling Technology	Cat#13586
anti-p-NF-κB p105 (Ser932)	Cell Signaling Technology	Cat#4806
anti-NF-κB p65	Cell Signaling Technology	Cat#4764
APC anti-human CD138 (Syndecan-1)	BioLegend	Cat#352308
APC Mouse IgG1, κ Isotype Ctrl	BioLegend	Cat#400120

Reference in supplemental Materials:

1. Huang X, Gu H, Zhang E, Chen Q, Cao W, Yan H, et al. The NEDD4-1 E3 ubiquitin ligase: A potential molecular target for bortezomib sensitivity in multiple myeloma. Int J Cancer. 2020;146:1963-1978.
2. Ren Z, Ahn JH, Liu H, Tsai YH, Bhanu NV, Koss B, et al. PHF19 promotes multiple myeloma tumorigenicity through PRC2 activation and broad H3K27me3 domain formation. Blood. 2019;134:1176-1189.
3. Borowicz S, Van Scoyk M, Avasarala S, Karuppusamy Rathinam MK, Tauler J, Bikkavilli RK, et al. The soft agar colony formation assay. J Vis Exp. 2014;10.3791/51998:e51998.
4. Jia G, Fu Y, Zhao X, Dai Q, Zheng G, Yang Y, et al. N6-methyladenosine in nuclear RNA is a major substrate of the obesity-associated FTO. Nat Chem Biol. 2011;7:885-887.
5. Kanehisa M, Goto S. KEGG: Kyoto Encyclopedia of Genes and Genomes. Nucleic Acids Res. 2000;28:27-30.
6. Dominissini D, Moshitch-Moshkovitz S, Schwartz S, Salmon-Divon M, Ungar L, Osenberg S, et al. Topology of the human and mouse m(6)A RNA methylomes revealed by m(6)A-seq. Nature. 2012;485:201-206.
7. Chen CYA, Ezzeddine N, Shyu AB. Messenger RNA half-life measurements in mammalian cells. Methods Enzymol. 2008;448:335-357.
8. Subramanian A, Tamayo P, Mootha VK, Mukherjee S, Ebert BL, Gillette MA, et al. Gene set enrichment analysis: a knowledge-based approach for interpreting genome-wide expression profiles. Proc Natl Acad Sci U S A. 2005;102:15545-15550.
9. Zhan F, Huang Y, Colla S, Stewart JP, Hanamura I, Gupta S, et al. The molecular classification of multiple myeloma. Blood. 2006;108:2020-2028.
10. Mulligan G, Mitsiades C, Bryant B, Zhan F, Chng WJ, Roels S, et al. Gene expression profiling and correlation with outcome in clinical trials of the proteasome inhibitor bortezomib. Blood. 2006;109:3177-3188.
11. Tarte K, Zhan F, De Vos J, Klein B, Shaughnessy J, Jr. Gene expression profiling of plasma cells and plasmablasts: toward a better understanding of the late stages of B-cell differentiation. Blood. 2003;102:592-600.
12. Chapman MA, Lawrence MS, Keats JJ, Cibulskis K, Sougnez C, Schinzel AC, et al. Initial genome sequencing and analysis of multiple myeloma. Nature. 2011;471:467-472.
13. Broyl A, Hose D, Lokhorst H, de Knegt Y, Peeters J, Jauch A, et al. Gene expression profiling for molecular classification of multiple myeloma in newly diagnosed patients. Blood. 2010;116:2543-2553.
14. Heuck CJ, Qu P, van Rhee F, Waheed S, Usmani SZ, Epstein J, et al. Five gene probes carry most of the discriminatory power of the 70-gene risk model in multiple myeloma. Leukemia. 2014;28:2410-2413.
15. Weinhold N, Ashby C, Rasche L, Chavan SS, Stein C, Stephens OW, et al. Clonal selection and double-hit events involving tumor suppressor genes underlie relapse in myeloma. Blood. 2016;128:1735-1744.
16. Dickens NJ, Walker BA, Leone PE, Johnson DC, Brito JL, Zeisig A, et al. Homozygous Deletion Mapping in Myeloma Samples Identifies Genes and an Expression Signature Relevant to Pathogenesis and Outcome. Clin Cancer Res. 2010;16:1856.

17. Zhan F, Barlogie B, Arzoumanian V, Huang Y, Williams DR, Hollmig K, et al. Gene-expression signature of benign monoclonal gammopathy evident in multiple myeloma is linked to good prognosis. Blood. 2006;109:1692-1700.
18. Todoerti K, Agnelli L, Fabris S, Lionetti M, Tuana G, Mosca L, et al. Transcriptional characterization of a prospective series of primary plasma cell leukemia revealed signatures associated with tumor progression and poorer outcome. Clin Cancer Res. 2013;19:3247-3258.
19. Kuo AJ, Cheung P, Chen K, Zee BM, Kioi M, Lauring J, et al. NSD2 links dimethylation of histone H3 at lysine 36 to oncogenic programming. Mol Cell. 2011;44:609-620.
20. Popovic R, Martinez-Garcia E, Giannopoulou EG, Zhang Q, Zhang Q, Ezponda T, et al. Histone methyltransferase MMSET/NSD2 alters EZH2 binding and reprograms the myeloma epigenome through global and focal changes in H3K36 and H3K27 methylation. PLoS Genet. 2014;10:e1004566.
21. Martinez-Garcia E, Popovic R, Min DJ, Sweet SMM, Thomas PM, Zamdborg L, et al. The MMSET histone methyl transferase switches global histone methylation and alters gene expression in t(4;14) multiple myeloma cells. Blood. 2011;117:211-220.
22. Drexler HG, Matsuo Y. Malignant hematopoietic cell lines: in vitro models for the study of multiple myeloma and plasma cell leukemia. Leuk Res. 2000;24:681-703.
23. Bolli N, Li Y, Sathiaseelan V, Raine K, Jones D, Ganly P, et al. A DNA target-enrichment approach to detect mutations, copy number changes and immunoglobulin translocations in multiple myeloma. Blood Cancer J. 2016;6:e467.
24. Surget S, Descamps G, Brosseau C, Normant V, Maiga S, Gomez-Bougie P, et al. RITA (Reactivating p53 and Inducing Tumor Apoptosis) is efficient against TP53abnormal myeloma cells independently of the p53 pathway. BMC Cancer. 2014;14:437.
25. Hanamura I, Stewart JP, Huang Y, Zhan F, Santra M, Sawyer JR, et al. Frequent gain of chromosome band 1q21 in plasma-cell dyscrasias detected by fluorescence in situ hybridization: incidence increases from MGUS to relapsed myeloma and is related to prognosis and disease progression following tandem stem-cell transplantation. Blood. 2006;108:1724-1732.
26. Bergsagel PL, Kuehl WM, Zhan F, Sawyer J, Barlogie B, Shaughnessy J, Jr. Cyclin D dysregulation: an early and unifying pathogenic event in multiple myeloma. Blood. 2005;106:296-303.
27. Smith D, Mann D, Yong K. Cyclin D type does not influence cell cycle response to DNA damage caused by ionizing radiation in multiple myeloma tumours. Br J Haematol. 2016;173:693-704.
28. Avet-Loiseau H, Gerson F, Magrangeas F, Minvielle Sp, Harousseau J-L, Bataille Rg, et al. Rearrangements of the c-myc oncogene are present in 15% of primary human multiple myeloma tumors. Blood. 2001;98:3082-3086.
29. Shou Y, Martelli ML, Gabrea A, Qi Y, Brents LA, Roschke A, et al. Diverse karyotypic abnormalities of the c-myc locus associated with c-myc dysregulation and tumor progression in multiple myeloma. Proc Natl Acad Sci U S A. 2000;97:228-233.
